# Associations of obesity and malnutrition with cardiac remodeling and cardiovascular outcomes in Asian adults: A cohort study

**DOI:** 10.1371/journal.pmed.1003661

**Published:** 2021-06-01

**Authors:** Shih-Chieh Chien, Chanchal Chandramouli, Chi-In Lo, Chao-Feng Lin, Kuo-Tzu Sung, Wen-Hung Huang, Yau-Huei Lai, Chun-Ho Yun, Cheng-Huang Su, Hung-I Yeh, Ta-Chuan Hung, Chung-Lieh Hung, Carolyn S. P. Lam

**Affiliations:** 1 Department of Critical Care Medicine, MacKay Memorial Hospital, Taipei, Taiwan; 2 Department of Medicine, Mackay Medical College, New Taipei City, Taiwan; 3 Division of Cardiology, Department of Internal Medicine, MacKay Memorial Hospital, Taipei, Taiwan; 4 National Heart Centre Singapore, Singapore; 5 Duke–NUS Medical School, Singapore; 6 MacKay Junior College of Medicine, Nursing, and Management, New Taipei City, Taiwan; 7 Department of Radiology, MacKay Memorial Hospital, Taipei, Taiwan; 8 University Medical Centre Groningen, Groningen, the Netherlands; 9 Institute of Biomedical Sciences, Mackay Medical College, New Taipei, Taiwan; 10 George Institute for Global Health, Sydney, New South Wales, Australia; George Institute for Global Health, AUSTRALIA

## Abstract

**Background:**

Obesity, a known risk factor for cardiovascular disease and heart failure (HF), is associated with adverse cardiac remodeling in the general population. Little is known about how nutritional status modifies the relationship between obesity and outcomes. We aimed to investigate the association of obesity and nutritional status with clinical characteristics, echocardiographic changes, and clinical outcomes in the general community.

**Methods and findings:**

We examined 5,300 consecutive asymptomatic Asian participants who were prospectively recruited in a cardiovascular health screening program (mean age 49.6 ± 11.4 years, 64.8% male) between June 2009 to December 2012. Clinical and echocardiographic characteristics were described in participants, stratified by combined subgroups of obesity and nutritional status. Obesity was indexed by body mass index (BMI) (low, ≤25 kg/m^2^ [lean]; high, >25 kg/m^2^ [obese]) (WHO-recommended Asian cutoffs). Nutritional status was defined primarily by serum albumin (SA) concentration (low, <45 g/L [malnourished]; high, ≥45 g/L [well-nourished]), and secondarily by the prognostic nutritional index (PNI) and Global Leadership Initiative on Malnutrition (GLIM) criteria. Cox proportional hazard models were used to examine a 1-year composite outcome of hospitalization for HF or all-cause mortality while adjusting for age, sex, and other clinical confounders. Our community-based cohort consisted of 2,096 (39.0%) lean–well-nourished (low BMI, high SA), 1,369 (25.8%) obese–well-nourished (high BMI, high SA), 1,154 (21.8%) lean–malnourished (low BMI, low SA), and 681 (12.8%) obese–malnourished (high BMI, low SA) individuals. Obese–malnourished participants were on average older (54.5 ± 11.4 years) and more often women (41%), with a higher mean waist circumference (91.7 ± 8.8 cm), the highest percentage of body fat (32%), and the highest prevalence of hypertension (32%), diabetes (12%), and history of cardiovascular disease (11%), compared to all other subgroups (all *p <* 0.001). N-terminal pro B-type natriuretic peptide (NT-proBNP) levels were substantially increased in the malnourished (versus well-nourished) groups, to a similar extent in lean (70.7 ± 177.3 versus 36.8 ± 40.4 pg/mL) and obese (73.1 ± 216.8 versus 33.2 ± 40.8 pg/mL) (*p <* 0.001 in both) participants. The obese–malnourished (high BMI, low SA) group also had greater left ventricular remodeling (left ventricular mass index, 44.2 ± 1.52 versus 33.8 ± 8.28 gm/m^2^; relative wall thickness 0.39 ± 0.05 versus 0.38 ± 0.06) and worse diastolic function (TDI-e′ 7.97 ± 2.16 versus 9.87 ± 2.47 cm/s; E/e′ 9.19 ± 3.01 versus 7.36 ± 2.31; left atrial volume index 19.5 ± 7.66 versus 14.9 ± 5.49 mL/m^2^) compared to the lean–well-nourished (low BMI, high SA) group, as well as all other subgroups (*p <* 0.001 for all). Over a median 3.6 years (interquartile range 2.5 to 4.8 years) of follow-up, the obese–malnourished group had the highest multivariable-adjusted risk of the composite outcome (hazard ratio [HR] 2.49, 95% CI 1.43 to 4.34, *p =* 0.001), followed by the lean–malnourished (HR 1.78, 95% CI 1.04 to 3.04, *p =* 0.034) and obese–well-nourished (HR 1.41, 95% CI 0.77 to 2.58, *p =* 0.27) groups (with lean–well-nourished group as reference). Results were similar when indexed by other anthropometric indices (waist circumference and body fat) and other measures of nutritional status (PNI and GLIM criteria). Potential selection bias and residual confounding were the main limitations of the study.

**Conclusions:**

In our cohort study among asymptomatic community-based adults in Taiwan, we found that obese individuals with poor nutritional status have the highest comorbidity burden, the most adverse cardiac remodeling, and the least favorable composite outcome.

## Abstract 2

Shih-Chieh-Chien and co-workers study cardiac outcomes in people with varying obesity and nutritional status in Taiwan.

## Author summary

**Why was this study done?:**

- Malnutrition is characterized by under- or overnutrition. Obesity, a condition with overnutrition, is an established risk factor for cardiovascular disease and cardiac failure.
- When undernutrition coexists alongside obesity (being overweight), a double burden of malnutrition is present.
- Whilst it is possible that malnutrition will worsen the cardiac effects of obesity, no study has examined this relationship in depth, particularly in Asia.
- We examined whether nutritional status (assessed using serum albumin [SA], prognostic nutritional index, or Global Leadership Initiative on Malnutrition criteria) affects the relationship between various obesity measures (body mass index [BMI], waist circumference, and body fat percentage) and cardiovascular outcomes in a general community population in Taiwan.

**What did the researchers do and find?:**

- We characterized asymptomatic adult participants into 4 combined subgroups of nutrition and obesity, namely, lean–well-nourished, lean–malnourished, obese–well-nourished, and obese–malnourished.
- The obese–malnourished (high BMI, low SA) individuals were typically older and often women, and had a higher likelihood of visceral obesity, comorbidities, notable cardiac remodeling, and impaired cardiac relaxation function, compared to all other groups.
- Obese–malnourished individuals also had the greatest risk of the composite outcome of heart failure hospitalization or all-cause mortality, compared to the lean–well-nourished (low BMI, high SA) group. Results were consistent across various measures of obesity and nutritional status.

**What do these findings mean?:**

- The double burden of malnutrition exists among Asian adults, whereby undernutrition is present along with obesity.
- Among Asian asymptomatic adults, obese individuals with coexisting malnutrition (obese–malnourished) have the highest comorbidity burden, maladaptive cardiac remodeling, and least favorable cardiac outcomes.

## Introduction

Rapid urbanization and industrialization in Asia in the past decades have led to an escalated prevalence of obesity in the region, especially among young adults in low- and middle-income countries [1]. Obesity is a known risk factor for cardiovascular disease and heart failure (HF) [2], including in Asia [3]. Obese individuals, with obesity typically defined using BMI, are also highly heterogenous, with a wide metabolic health spectrum [4]. Among the metabolically unhealthy individuals, obesity coexists with multiple comorbidities (such as hypertension, diabetes, dyslipidemia, and sleep apnea), entailing deleterious systemic and cardiac effects. These include cardiac remodeling, subclinical myocardial dysfunction, HF with preserved ejection fraction (HFpEF), and coronary artery disease [5]. On the other hand, some individuals remain metabolically healthy with obesity. While body composition and fat distribution may explain some aspects of the heterogeneity in obese individuals, there remains a strong need to explain the varying predispositions towards metabolic disturbances and the risk of cardiac complications [5].

Malnutrition is any nutritional imbalance resulting in under- or overnutrition. The World Health Organization (WHO) has highlighted “a double burden of malnutrition”—characterized by the coexistence of undernutrition and being overweight or obese or having diet-related non-communicable diseases—as a real and growing global health challenge [6]. Serum albumin (SA) and prognostic nutritional index (PNI) are surrogate markers of nutritional status [7–9], vital for physiological function and shown to be prognostic for outcomes in multiple cardiovascular diseases, particularly HF [10–12]. It is likely that malnutrition may mediate/exacerbate cardiac effects associated with chronic morbid obesity. To our knowledge, no study has examined the combined effects of obesity and malnutrition on cardiac outcomes in an asymptomatic population. Previous studies have focused on critically ill patients [13,14], predominantly relied on BMI as the sole surrogate measure of obesity [14], or utilized inconsistent measures of nutrition [15,16]. In this study, we aimed to investigate whether nutritional status (SA and PNI) modifies the relationship between various indices of obesity (BMI, waist circumference, and body composition) and clinical characteristics, cardiac remodeling, and outcomes in the general population. We report that obesity significantly modified the relationship between nutritional status and key diastolic indices.

## Methods

### Study setting and population

The cohort study design, inclusion/exclusion criteria, and outcomes of the cardiovascular health screening program have been published previously [17–19]. Briefly, the study population consisted of 5,527 consecutive participants in a cardiovascular health screening program (from June 2009 to December 2012) at a tertiary medical center in northern Taipei, Taiwan. After excluding participants with advanced cardiac conditions (atrial fibrillation, prevalent or known history of HF, significant valvular heart diseases, congenital heart disease, or hypertrophic cardiomyopathy) and poor echocardiography images, 5,300 participants were finally included in the analysis. All patients underwent a detailed physical examination at baseline, which included BMI, waist circumference, and bioelectrical-impedance-based body composition analysis (Tanita TBF-305 body fat analyzer, Tanita, Tokyo, Japan). A structured 260-item questionnaire was used to obtain baseline clinical information, medical history, symptoms, signs, and lifestyle factors. Ethics approval was obtained from the relevant local human ethics committee at the MacKay Memorial Hospital (16MMHIS142e, 19 April 2018). This study complied with the Declaration of Helsinki, and all patients provided written informed consent (S1 Checklist STROBE).

### Nutrition and blood measurements

Blood samples were obtained after an overnight fast, in a sitting position. SA concentration was measured using an automatic biochemistry analyzer (Hitachi 7170, Hitachi, Tokyo, Japan). Serum levels of hemoglobin A1c (HbA1c), aminotransferases, high-sensitivity C-reactive protein (hs-CRP), creatinine, glucose, uric acid, and N-terminal pro B-type natriuretic peptide (NT-proBNP); lipid panel (total cholesterol, low-density lipoprotein cholesterol [LDL-C], high-density lipoprotein cholesterol [HDL-C], triglycerides); and blood count were measured using standard laboratory techniques. NT-proBNP level was measured in 4,840 (91.3%) participants using an electrochemiluminescence immunoassay (Roche E170, Roche Diagnostics, Mannheim, Germany). hs-CRP was available for 3,755 (71%) participants and was assessed using a highly sensitive latex-particle-enhanced immunoassay method (Elecsys 2010, Roche Diagnostics, Mannheim, Germany). PNI, a surrogate marker of nutritional status, was computed with the following formula: SA level (g/L) + 0.005 × total lymphocyte count (per mm^3^) [12].

### Standard echocardiography protocol

Echocardiography at baseline was uniformly performed using the GE system (Vivid i) equipped with a 2- to 4-MHz transducer (3S-RS) according to internationally accepted guidelines [20]. The core laboratory of echocardiography within MacKay Memorial Hospital provided oversight, imaging protocol guidelines, and quality assurance of echocardiograms. The standard echocardiographic protocol included an assessment of the left ventricular (LV) dimensions (diameter, wall thickness, and volume during systole and diastole), LV ejection fraction (LVEF), left atrial dimensions, and echo doppler estimates of LV diastolic function [20]. Accuracy and reproducibility of the interpreted results were ensured through consistent training and systematic analytical processes, as specified in international guidelines [20]. LV mass was calculated from linear dimensions and indexed to body surface area (BSA) or height (Ht^2.7^). Relative wall thickness (RWT) was calculated by the formula (2 × diastolic posterior wall thickness)/diastolic LV internal diameter. LV hypertrophy (LVH) was determined as the LV mass indexed to BSA > 115 g/m^2^ in men and BSA > 95 g/m^2^ in women. Normal LV geometry was defined as having no LVH and an RWT ≤ 0.42. Abnormal LV geometry was classified as concentric remodeling (no LVH and RWT > 0.42), concentric hypertrophy (LVH and RWT > 0.42), or eccentric hypertrophy (LVH and RWT ≤ 0.42). The left atrial size was indexed to height [20]. The LV diastolic function was determined by pulsed-wave doppler of early (E) and late diastolic (A) LV inflow velocities at the tip of the mitral leaflets utilizing the LV apical 4-chamber view, with tissue Doppler imaging (TDI)–based peak myocardial relaxation velocity (TDI-e′) obtained at the basal septal and lateral mitral annular regions using a high-frame-rate spectral Doppler. The LV filling pressure was estimated using the noninvasive E/e′ ratio [21].

### Study definitions

Nutritional status was primarily defined by SA concentration (g/L) in 5,300 individuals (low SA, <45 g/L [malnourished]; high SA, ≥45 g/L [well-nourished]). Secondary analysis was conducted among 4,982 individuals who also had total lymphocyte count available with nutritional status indexed by PNI, a surrogate indicator of nutritional and immune status (low PNI, <55 [malnourished]; high PNI, ≥55 [well-nourished]). Our prespecified analyses included defining nutrition by SA and PNI, which are shown to be important malnutrition markers with prognostic HF outcomes [10–12]. Following suggestions during the peer-review process, we conducted an additional sensitivity analysis defining malnutrition by the Global Leadership Initiative on Malnutrition (GLIM)–recommended criteria [22]. For this analysis, malnutrition was defined as any of the phenotypic criteria (1) patient-reported weight loss of at least 5 kg in the last 6 months, (2) low body mass index (<18.5 kg/m^2^, based on Asian cutoff), or (3) reduced muscle mass (free fat mass < 15.6 kg and <13.7 kg in men and women, respectively), in combination with an etiological criterion of inflammation (hs-CRP level > 3.0 mg/L). The population- and sex-specific cutoffs for reduction in muscle mass were determined at 2 standard deviations below the mean for young, healthy Taiwanese adults, as described in other studies [23–25].

Obesity was defined according to standard body mass index (BMI) as defined by the World Health Organization (WHO) (Asian cutoff, ≥25 kg/m^2^) [26]. For secondary analyses, we also defined obesity using sex-specific cutoffs for waist circumference (>90 cm and >80 cm in men and women, respectively) [27] or percentage body fat (>25% and >35% in men and women, respectively) [28]. Estimated glomerular filtration rate (eGFR) was calculated using the Modification of Diet in Renal Disease (MDRD) study equation [29].

### Outcomes

Individuals were prospectively followed up for a median of 3.6 years (IQR 2.5 to 4.8 years). The primary outcome examined was a composite outcome of HF hospitalization and all-cause mortality at 1 year. Outcomes were adjudicated by 2 independent cardiologists reviewing clinical notes and medical records. All data were documented in an electronic database capture system, which was managed by the core echocardiography laboratory appointed by MacKay Memorial Hospital.

### Statistical analysis

Participants were categorized into 4 groups according to their BMI (high, >25 kg/m^2^ [obese]; low, ≤25 kg/m^2^ [lean]) and SA concentrations (low, <45 g/L [malnourished]; high, ≥45 g/L [well-nourished]). Continuous data are summarized as mean ± standard deviation (normal distribution) or median with interquartile range (IQR) (non-normal distribution), and categorical data are expressed as proportions. The differences between BMI/SA groups were tested using ANOVA for continuous variables with post hoc paired comparisons with Bonferroni corrections (Table 1). As secondary analyses, obesity was also indexed by (1) waist circumference (high, >90 cm or >80 cm, and low, ≤90 cm or ≤80 cm, in men and women, respectively) [28] (S1 Table) and (2) percentage body fat (high, >25% and >35%, and low, ≤25% and ≤35%, in men and women, respectively) [27] (S2 Table).

**Table 1:** Baseline demographics of study participants according to obesity and serum albumin categories.

Characteristic	Lean–well-nourished (BMI ≤ 25 kg/m^2^, SA ≥ 45 g/L)	Obese–well-nourished (BMI > 25 kg/m^2^, SA ≥ 45 g/L)	Lean–malnourished (BMI ≤ 25 kg/m^2^, SA < 45 g/L)	Obese–malnourished (BMI > 25 kg/m^2^, SA < 45 g/L)	*p-*Value
Total *n* (*N* = 5,300)	2,096 (39%)	1,369 (25.8%)	1,154 (21.8%)	681 (12.8%)	
*Demographic data*					
Age, years	47.2 ± 11.5	48.2 ± 10.2	52.6 ± 11.5*^#^	54.5 ± 11.4*^#^^†^	<0.001
Sex, male	1,386 (66.1%)	1,180 (86.2%)	486 (42.1%)	404 (59.3%)	<0.001
Body mass index, kg/m^2^	22.2 ± 1.91	27.8 ± 2.59^#^	22.2 ± 1.88^#^	28.2 ± 3.21*^#^^†^	<0.001
Systolic blood pressure, mm Hg	120.2 ± 16.1	128.5 ± 16.7^#^	119.2 ± 17.3^#^	128.5 ± 18.4*^†^	<0.001
Heart rate, beats/min	67.3 ± 11.0	69.0 ± 11.5^#^	66.1 ± 11.2*^#^	67.8 ± 12.4^†^	<0.001
Body fat, percent	23.3 ± 5.1	29.2 ± 5.97^#^	24.5 ± 5.75*^#^	31.9 ± 8.65*^#^^†^	<0.001
Waist circumference, cm	78.7 ± 7.21	91.8 ± 7.60^#^	77.8 ± 7.54*^#^	91.7 ± 8.81^†^	<0.001
Fat mass, percent	14.5 ± 3.49	23.2 ± 6.30^#^	14.4 ± 3.66^#^	24.1 ± 8.34*^#^^†^	<0.001
Fat-free mass, percent	47.5 ± 7.72	55.8 ± 8.97^#^	44.0 ± 7.49*^#^	51.5 ± 9.74*^#^^†^	<0.001
Hypertension	262 (12.5%)	351 (25.6%)	166 (14.4%)	212 (31.1%)	<0.001
Diabetes	86 (4.1%)	110 (8.0%)	86 (7.5%)	87 (12.8%)	<0.001
Cardiovascular disease	109 (5.2%)	100 (7.3%)	96 (8.3%)	76 (11.2%)	<0.001
Smoking	171 (8.2%)	164 (12.0%)	133 (11.5%)	93 (13.7%)	<0.001
Exercise	261 (12.5%)	188 (13.7%)	182 (15.8%)	97 (14.2%)	0.07
*Laboratory data and biomarkers*					
White blood count, ×10^9^/L	5.90 ± 1.48	6.63 ± 1.66^#^	5.79 ± 1.59^#^	6.53 ± 1.74*^†^	<0.001
Fasting glucose, mmol/L	5.43 ± 0.87	5.91 ± 1.41^#^	5.46 ± 1.24^#^	5.89 ± 1.44*^†^	<0.001
eGFR, mL/min/1.73 m^2^	89.2 ± 16.2	85.9 ± 15.1^#^	90.9 ± 19.6^#^	88.5 ± 17.5^†^	<0.001
Total cholesterol, mmol/L	5.2 ± 0.9	5.3 ± 0.9^#^	5.1 ± 1.0*^v^	5.2 ± 0.9^#^	<0.001
Triglycerides, mmol/L	1.3 ± 1.0	1.9 ± 1.2^#^	1.3 ± 1.5^#^	1.7 ± 1.0*^#^^†^	<0.001
LDL-C, mmol/L	3.3 ± 0.9	3.5 ± 0.8^#^	3.2 ± 0.9*^#^	3.4 ± 0.8^#^^†^	<0.001
HDL-C, mmol/L	1.5 ± 0.4	1.2 ± 1.5^#^	1.5 ± 0.4^#^	1.3 ± 0.3^#^^†^	<0.001
Total protein, g/L	75.4 ± 3.7	75.8 ± 3.7^#^	72.0 ± 4.1*^#^	73.0 ± 3.7*^#^^†^	<0.001
Serum GPT, U/L	25.2 ± 16.3	40.7 ± 31.0^#^	23.2 ± 23.0^#^	33.5 ± 29.8*^#^^†^	<0.001
NT-proBNP, pg/mL	36.8 ± 40.4	33.2 ± 40.8	70.7 ± 177.3*^#^	73.1 ± 216.8*^#^	<0.001
hs-CRP, mg/L	1.4 ± 3.2	2.4 ± 3.6^#^	2.0 ± 4.2*	2.9 ± 3.7*^†^	<0.001

BMI, body mass index; eGFR, estimated glomerular filtration rate; GPT, glutamic pyruvic transaminase; HDL-C, high-density lipoprotein cholesterol; hs-CRP, high-sensitivity C-reactive protein; LDL-C, low-density lipoprotein cholesterol; NT-proBNP, N-terminal pro B-type natriuretic peptide; SA, serum albumin.

Data are given as mean ± standard deviation or number (percent). *p*-Value < 0.05 for comparison against *lean–well-nourished

^†^lean–malnourished, and

^#^obese–well-nourished.

Echocardiographic data are presented as unadjusted and multivariable-adjusted means with *p*-values estimated from linear regression for the trend across categories. Adjusted models included age, sex, systolic blood pressure, heart rate, fasting glucose, HDL-C, total cholesterol, hypertension, diabetes, cardiovascular disease, and eGFR. Differences between the 4 subgroups were tested using ANOVA for continuous variables with post hoc paired comparisons with Bonferroni corrections (Table 2). Based on the recommendations during the peer-review process, the associations between SA level (as a continuous variable) and echocardiographic indices were examined by linear regression with interaction tests for BMI to investigate the possible modifying effects of obesity. When significant interactions were present, stratified analysis was performed for BMI (lean and obese) (S4 Table).

**Table 2:** Echocardiography information of study participants according to obesity and serum albumin categories.

Measure	Non-adjusted mean ± SD					Multivariable-adjusted mean				
	Lean–well-nourished (BMI ≤ 25 kg/m^2^, SA ≥ 45 g/L)	Obese–well-nourished (BMI > 25 kg/m^2^, SA ≥ 45 g/L)	Lean–malnourished (BMI ≤ 25 kg/m^2^, SA < 45 g/L)	Obese–malnourished (BMI > 25 kg/m^2^, SA < 45 g/L)	*p* trend	Lean–well-nourished (BMI ≤ 25 kg/m^2^, SA ≥ 45 g/L)	Obese–well-nourished (BMI > 25 kg/m^2^, SA ≥ 45 g/L)	Lean–malnourished (BMI ≤ 25 kg/m^2^, SA < 45 g/L)	Obese–malnourished (BMI > 25 kg/m^2^, SA < 45 g/L)	*p* trend
*N*	2,096	1,154	1,369	681		2,096	1,154	1,369	681	
IVERSUS, mm	8.74 ± 1.24	9.48 ± 1.28*	8.78 ± 1.23^#^	9.59 ± 1.27*^†^	<0.001	8.85	8.92*	9.28^#^	9.36^#^^†^	<0.001
LVPW, mm	8.72 ± 1.14	9.44 ± 1.13*	8.75 ± 1.14^#^	9.59 ± 1.22*^†^	<0.001	8.82	8.87*	9.25^#^	9.35*^†^	<0.001
IVERSUSi (Ht^2.7^), mm/m^2^	4.85 ± 0.73	4.63 ± 0.70*	5.08 ± 0.76*^#^	4.86 ± 0.74^#^^†^	<0.001	4.91	4.94*	4.69^#^	4.68^#^^†^	<0.001
LVPWi (Ht^2.7^), mm/m^2^	4.84 ± 0.67	4.61 ± 0.62*	5.06 ± 0.72*^#^	4.86 ± 0.71^#^^†^	<0.001	4.89	4.92*	4.67^#^	4.68^#^^†^	<0.001
LVIDD, mm	45.9 ± 3.67	48.4 ± 3.16*	45.9 ± 3.85^#^	48.4 ± 3.29*^†^	<0.001	46.1	46.5*^#^	47.8^#^	48.2*^#^^†^	<0.001
LVIDS, mm	28.9 ± 2.91	30.5 ± 2.84*	28.7 ± 3.01^#^	30.5 ± 3.14*^†^	<0.001	28.9	29.1*	30.0^#^	30.4*^#^^†^	<0.001
LV EDV, mL	73.1 ± 13.9	82.6 ± 12.9*	72.3 ± 13.8^#^	81.9 ± 12.8*^†^	<0.001	73.4	75.1*^#^	80.2^#^	82.0*^#^^†^	<0.001
LV ESV, mL	27.4 ± 7.11	31.4 ± 7.75*	26.7 ± 7.06^#^	30.9 ± 7.46*^†^	<0.001	27.4	28.0*	30.2^#^	30.9*^#^^†^	<0.001
LV EDVi (Ht^2.7^), mL/m^2^	40.3 ± 6.52	40.2 ± 5.96	41.6 ± 7.03*^#^	41.5 ± 6.42*^#^	<0.001	40.4	41.4*	40.3^#^	41.1*^#^^†^	<0.001
LV ESVi (Ht^2.7^), mL/m^2^	15.0 ± 3.48	15.3 ± 3.67	15.4 ± 3.74	15.6 ± 3.69*	<0.001	15.1	15.4^#^	15.1	15.5^#^	<0.001
LVEF, percent	62.7 ± 5.33	62.2 ± 5.62	63.2 ± 5.54^#^	62.4 ± 5.65^†^	0.25	62.8	62.9	62.5	62.4	0.53
LV mass, gm/m^2^	133.3 ± 32.6	161.0 ± 35.1*	134.1 ± 34.1^#^	164.4 ± 36.9*^†^	<0.001	136.4	139.8*^#^	154.1^#^	158.3*^#^^†^	<0.001
LV mass index (BSA), gm/m^2^	73.4 ± 16.3	78.4 ± 16.4*	77.0 ± 18.3*	83.1 ± 19.0*^#^^†^	<0.001	75.0	76.9^#^	77.3*	79.0*^#^^†^	<0.001
LV mass index (Ht^2.7^), gm/m^2^	33.8 ± 8.28	39.9 ± 9.43*	36.6 ± 9.33*^#^	44.2 ± 1.52*^#^^†^	<0.001	35.0	35.9*	39.7*^#^	41.3*^#^^†^	<0.001
LVH, percent	81 (3.9%)	84 (6.1%)	102 (8.8%)	99 (14.5%)	<0.001	5.5%	6.1%	6.7%	9.5%*^#^^†^	0.003
RWT	0.38 ± 0.06	0.39 ± 0.05*	0.38 ± 0.05^#^	0.40 ± 0.06*^†^	<0.001	0.38	0.38*	0.39	0.39	<0.001
Geometry, number (%)					<0.001					<0.001
Normal	1,696 (81%)	1,050 (76.8%)	878 (76.1%)	474 (69.6%)	—	—	—	—	—	
Concentric remodeling	318 (15.2%)	234 (17.1%)	174 (15.1%)	108 (15.9%)	—	—	—	—	—	
Eccentric hypertrophy	23 (1.1%)	29 (2.1%)	57 (4.9%)	43 (6.3%)	—	—	—	—	—	
Concentric hypertrophy	58 (2.8%)	55 (4.0%)	45 (3.9%)	56 (8.2%)	—	—	—	—	—	
Deceleration time, ms	204.1 ± 39.0	204.8 ± 38.6*	204.7 ± 39.4	208.2 ± 42.7	0.09	202.3	203.4	205.0^#^	204.8	0.06
IVRT, ms	88.8 ± 14.3	90.8 ± 15.3	89.9 ± 15.2	92.7 ± 17.8*^†^	<0.001	89.8	90.0	90.2	91.4^†^	0.07
TDI-e′ (average), cm/s	9.87 ± 2.47	8.61 ± 2.08*	9.20 ± 2.42*^#^	7.97 ± 2.16*^#^^†^	<0.001	9.48	9.38*	8.79^#^	8.83^#^^†^	<0.001
TDI-s′ (average), cm/s	8.52 ± 1.59	8.28 ± 1.52*	8.10 ± 1.54*^#^	7.79 ± 1.43*^#^^†^	<0.001	8.40	8.33	8.21^#^	8.11^#^^†^	<0.001
E/A ratio	1.30 ± 0.44	1.13 ± 0.36*	1.26 ± 0.46*^#^	1.09 ± 0.42*^†^	<0.001	1.24	1.24*	1.17^#^	1.21*^†^	<0.001
E/e′ (average)	7.36 ± 2.31	8.04 ± 2.48*	8.27 ± 2.77*	9.19 ± 3.01*^#^^†^	<0.001	7.66	7.87*^#^	8.10^#^	8.44*^#^^†^	<0.001
Tau	38.6 ± 8.68	39.3 ± 8.69	41.1 ± 9.94*^#^	42.5 ± 10.6*^#^^†^	<0.001	39.1	39.5*	40.4^#^	41.5*^#^^†^	<0.001
TRV, m/s	2.09 ± 0.33	2.09 ± 0.34	2.18 ± 0.38*^#^	2.23 ± 0.36*^#^^†^	<0.001	2.10	2.13	2.11	2.18*^#^^†^	<0.001
LAV (max), mL	27.1 ± 9.98	36.1 ± 13.3*	29.7 ± 12.1*^#^	38.4 ± 14.3*^#^^†^	<0.001	27.5	29.6*^#^	34.9^#^	36.1*^#^^†^	<0.001
LAVi, mL/m^2^	14.9 ± 5.49	17.6 ± 6.35*	17.2 ± 7.09*	19.5 ± 7.66*^#^^†^	<0.001	15.2	16.3*^#^	17.5^#^	18.1^#^^†^	<0.001

BSA, body surface area; E/A ratio, ratio of early (E) to late (A) ventricular filling velocity; E/e′, ratio of early mitral inflow velocity to mitral annular early diastolic velocity; EDV, end diastolic volume; EDVi, end diastolic volume index; ESV, end systolic volume; ESVi, end systolic volume index; Ht, height; IVERSUS, interventricular septal wall thickness; IVERSUSi, interventricular septal wall thickness index; IVRT, interventricular relaxation time; LAV, left atrial volume; LAVi, left atrial volume index; LV, left ventricular; LVEF, left ventricular ejection fraction; LVH, left ventricular hypertrophy; LVIDD, left ventricular internal diameter in diastole; LVIDS, left ventricular internal diameter in systole; LVPW, left ventricular posterior wall thickness; LVPWi, left ventricular posterior wall thickness index; RWT, relative wall thickness; tau, left ventricular diastolic time constant; TDI-e′, tissue Doppler imaging–based peak myocardial relaxation velocity; TDI-s′, tissue Doppler imaging–based peak systolic annular velocity; TR, tricuspid regurgitation velocity.

Multivariable analysis adjusted for age, sex, systolic blood pressure, heart rate, fasting glucose, high-density lipoprotein cholesterol, total cholesterol, hypertension, diabetes, cardiovascular disease, and estimated glomerular filtration rate (eGFR).

*p*-Value < 0.05 for ANOVA post hoc (Bonferroni) comparison against *lean–well-nourished

^†^lean–malnourished, and

^#^obese–well-nourished.

The time to event analyses were performed using Cox proportional hazard models in the absence of violation of the proportional hazard assumption. The adjustments included age, sex, systolic blood pressure, heart rate, fasting glucose, HDL-C, total cholesterol, hypertension, diabetes, cardiovascular disease, and eGFR. The primary endpoint of the 1-year composite outcome (HF hospitalization or all-cause mortality) was censored at 1 year in patients who did not have an event (Table 3).

**Table 3:** Association of subgroups of obesity measures and SA with the composite outcome (heart failure hospitalization and all-cause mortality) (*n* = 145).

Subgroup	Crude events (*n*)	*p*_interaction_ with SA	Univariate HR (95% CI)	*p*-Value	Multivariate HR (95% CI)	*p*-Value
*Combined subgroups of BMI and SA*		0.75				
Lean–well-nourished	26		Reference		Reference	
Lean–malnourished	26		1.46 (0.84, 2.53)	0.18	1.41 (0.77, 2.58),	0.27
Obese–well-nourished	47		3.04 (1.88, 4.91)	**<0.001**	1.78 (1.04, 3.04)	**0.034**
Obese–malnourished	46		5.07 (3.13, 8.22)	**<0.001**	2.49 (1.43, 4.34)	**0.001**
*Combined subgroups of WC and SA*		0.62				
Low WC–well-nourished	27		Reference		Reference	
Low WC–malnourished	26		2.35 (1.36, 4.04)	**0.002**	1.78 (0.98, 3.24)	0.059
High WC–well-nourished	49		3.41 (2.13, 5.46)	**<0.001**	1.87 (1.08, 3.23)	**0.025**
High WC–malnourished	45		6.34 (3.93, 10.23)	**<0.001**	2.94 (1.65, 5.25)	**<0.001**
*Combined subgroups of BF and SA*		0.37				
Low BF–well-nourished	25		Reference		Reference	
Low BF–malnourished	24		1.58 (0.90, 2.78)	0.11	1.62 (0.88, 2.98)	0.12
High BF–well-nourished	55		3.55 (2.21, 5.70)	**<0.001**	2.01 (1.22, 3.34)	**0.007**
High BF–malnourished	26		3.82 (2.20, 6.63)	**<0.001**	2.20 (1.21, 4.01)	**0.01**

BF, body fat; HR, hazard ratio; SA, serum albumin; WC, waist circumference.

Multivariate HR adjusted for age, sex, systolic blood pressure, heart rate, fasting glucose, high-density lipoprotein cholesterol, total cholesterol, hypertension, diabetes, cardiovascular disease, and estimated glomerular filtration rate. Statistically significant *p*-values are in bold.

Patients with incomplete data on adjusted baseline characteristics were excluded from multivariable linear and Cox regression analyses. All analyses were performed using Stata (version 12, StataCorp, College Station, TX, US). The 2-tailed alpha significance level was set at 0.05 for all analyses, with a *p*-value of less than 0.05 being considered statistically significant.

## Results

### Baseline characteristics

Among 5,300 asymptomatic Taiwanese individuals (mean age 49.6 ± 11.4 years, 64.8% male), the mean BMI, SA level, and PNI were 24.4 ± 3.6 kg/m^2^, 45 ± 3 g/L, and 55.0 ± 4.2, respectively. Grouped by BMI and SA, there were 2,096 (39.0%) lean–well-nourished (low BMI, high SA), 1,369 (25.8%) obese–well-nourished (high BMI, high SA), 1,154 (21.8%) lean–malnourished (low BMI, low SA), and 681 (12.8%) obese–malnourished (high BMI, low SA) individuals (Table 1).

The lean–well-nourished (low BMI, high SA) individuals were the youngest participants on average, with a normal average BMI, the lowest percentage body fat (23.3% ± 5.1%), the fewest comorbidities (hypertension, diabetes, and cardiovascular disease), and the lowest prevalence of smoking (*p <* 0.001 for all; Table 1).

The lean–malnourished (low BMI, low SA) group had highest number women (57.9%) of all groups. Compared to their well-nourished counterparts, the lean–malnourished were approximately 5 years older on average (52.6 versus 47.2 years), with a higher percentage body fat (24.5% versus 23.3%) (despite similar BMI) and a greater comorbidity burden, especially diabetes (7.5% versus 4.1%) and cardiovascular disease (8.3% versus 5.2%) (*p <* 0.001 for all; Table 1).

The obese–well-nourished (high BMI, high SA) group had a predominance of men (86.2%) with high waist circumference on average, a higher percentage body fat (29.2%), and greater prevalence of hypertension (25.6%) (Table 1).

In contrast, the obese–malnourished (high BMI, low SA) individuals were the oldest participants on average (54.5 ± 11.4 years), more often women (40.7%), with high waist circumference (91.7 ± 8.8 cm), and the highest prevalence of hypertension (31.1%), diabetes (12.8%), cardiovascular diseases (11.2%), and smoking (13.7%), compared to all other subgroups (*p <* 0.001 for all). The obese–malnourished group also had the highest BMI (28.2 kg/m^2^ versus 27.8 kg/m^2^), percentage body fat (31.9%), and fat mass (24.1%), despite comparable waist circumference to the obese–well-nourished group (91.7 versus 91.8 cm) (*p* > 0.05; Table 1). Results were consistent when BMI was substituted with waist circumference (S1 Table) and percentage body fat (S2 Table).

### Biomarkers

**Fig 1:**
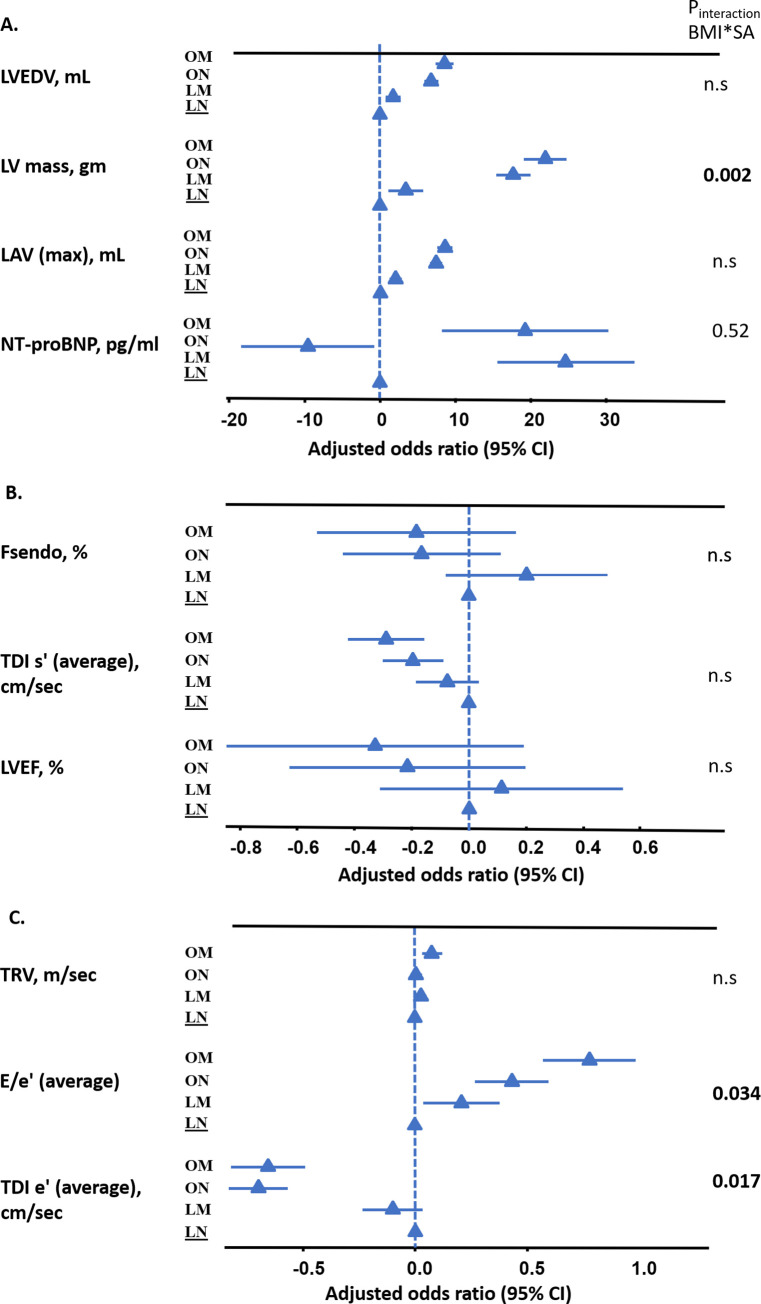
Forest plots depicting multivariable associations of key echocardiography measures and combined subgroups of BMI and nutrition. (A) Cardiac structural parameters; (B) LV systolic function; (C) LV diastolic function. Models adjusted for age, sex, systolic blood pressure, heart rate, fasting glucose, high-density lipoprotein cholesterol, total cholesterol, hypertension, diabetes, cardiovascular disease, and estimated glomerular filtration rate. E/e′, ratio of early mitral inflow velocity to mitral annular early diastolic velocity; Fsendo, endocardial fractional shortening; LAV, left atrial volume; LM, lean–malnourished; LN, lean–well-nourished; LV, left ventricular; LVEDV, left ventricular end diastolic volume; LVEF, left ventricular ejection fraction; NT-proBNP, N-terminal pro B-type natriuretic peptide; OM, obese–malnourished; ON, obese–well-nourished; SA, serum albumin; TDI e′, tissue Doppler imaging–based peak myocardial relaxation velocity; TDI s′, tissue Doppler imaging–based peak systolic annular velocity; TRV, tricuspid regurgitation velocity.

Biomarker NT-proBNP (73.1 ± 216.8 pg/mL) and hs-CRP (2.9 ± 3.7 mg/L) levels were highest in the obese–malnourished group, compared to all other subgroups. Of note, NT-proBNP levels were nearly double in both malnourished (versus well-nourished) groups, and were increased to a similar extent in both lean (70.7 versus 36.8 pg/mL), and obese participants (73.1 versus 33.2 pg/mL) (*p <* 0.001 for both; Table 1). Both obese–malnourished and lean–malnourished individuals showed significantly higher NT-proBNP levels, even after adjustment for confounders (Fig 1A).

### Echocardiography

Compared to the lean–well-nourished (low BMI, high SA) participants, the obese–well-nourished (high BMI, high SA) participants had greater left ventricular mass index (LVMi), LV wall thickness, and diastolic dysfunction (E/e′ 8.04 ± 2.48 versus 7.36 ± 2.31 and left atrial volume index [LAVi] 17.6 ± 6.35 versus 14.9 ± 5.49 mL/m^2^). Likewise, the lean–malnourished (low BMI, low SA) participants showed similar echocardiographic characteristics (greater LVMi, more diastolic dysfunction) when compared to their lean–well-nourished counterparts (Table 2).

The obese–malnourished individuals (high BMI, low SA) had the greatest LV remodeling (increased LVMi [indexed to BSA and height] and increased RWT), larger LV volume, and reduced diastolic function (TDI-e′ 7.97 ± 2.16 cm/s versus 9.87 ± 2.47 cm/s; E/e′ 9.19 ± 3.01 versus 7.36 ± 2.31; LAVi 19.50 ± 7.6 mL/m^2^ versus 14.9 ± 5.49 mL/m^2^; *p <* 0.001 for all) compared to the lean–well-nourished individuals (low BMI, high SA), as well as all other subgroups (Table 2; Fig 1). These differences were consistent even after adjusting for clinical confounders (age, sex, systolic blood pressure, heart rate, fasting glucose, HDL-C, total cholesterol, hypertension, diabetes, cardiovascular disease, and eGFR) (Table 2). Multivariable linear analysis showed that being obese–malnourished (high BMI, low SA) was consistently associated with greater diastolic dysfunction (average E/e′ 0.78, 95% CI 0.57 to 0.98), with the lean–well-nourished group as reference (Fig 1).

When SA was analyzed on a continuous scale, the analysis showed that lower SA concentrations were associated with poorer diastolic functional indices, even after multivariable correction (higher LA volume and lower TDI-e′; S4 Table). Increased concentrations of SA were associated with better diastolic function. These relationships were modified by BMI, waist circumference, and body fat categories (*p*_interaction_ < 0.05 for all; S4 Table). When stratified by tertiles, individuals with BMI ≥ 27.5 kg/m^2^ or with large waist circumference (≥80 cm in women and ≥90 cm in men) or high body fat composition (≥32% in women and ≥25% in men) had diastolic dysfunction (higher LA volumes), irrespective of SA level (S4 Table).

### Outcomes

**Fig 2:**
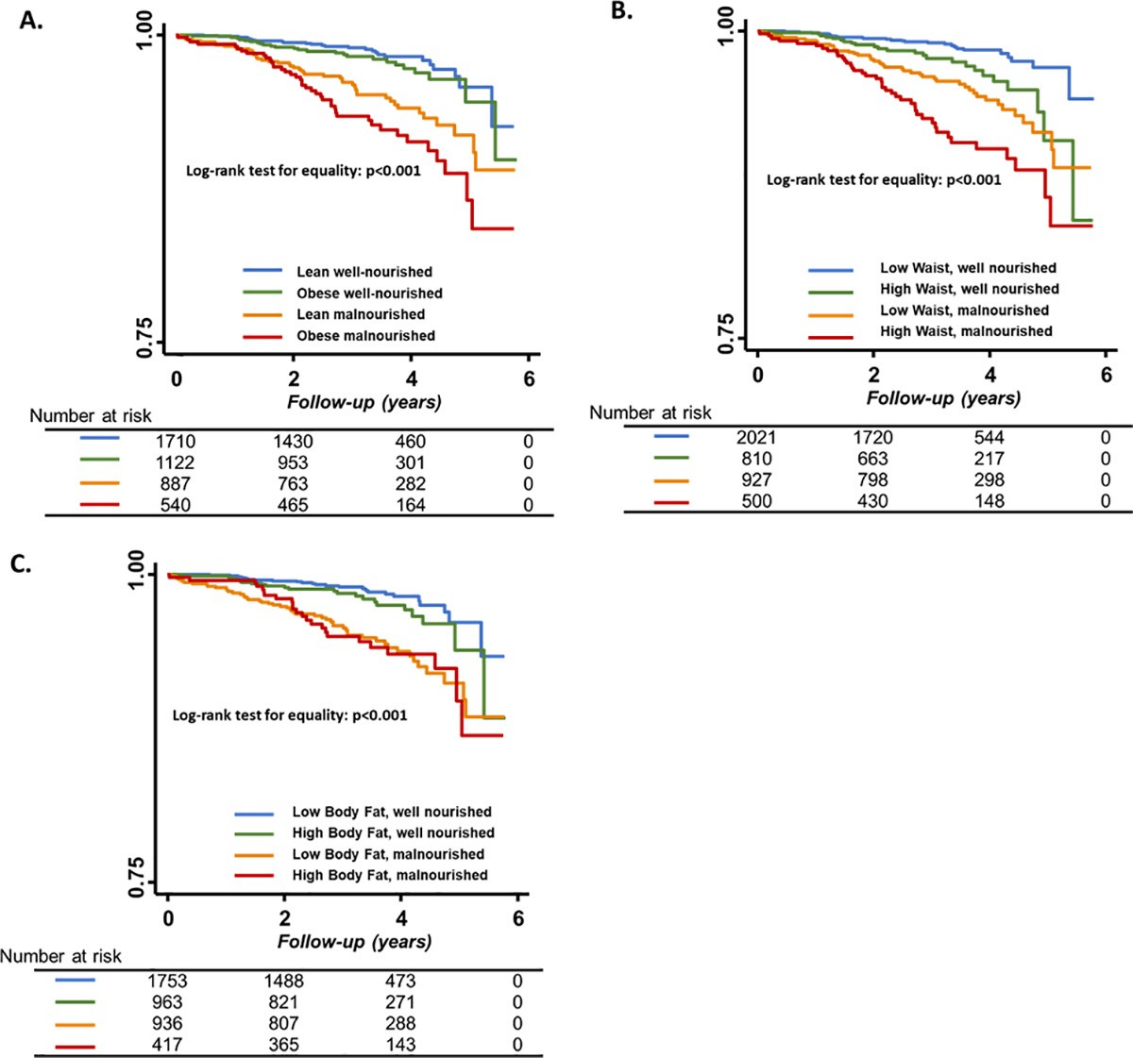
Kaplan–Meier survival estimates for composite outcome of heart failure hospitalization and 1-year mortality. Participants are stratified by combined subgroups of nutritional index (serum albumin) and (A) body mass index (high, >25 kg/m^2^), or (B) waist circumference (high, >90 cm and >80 cm in men and women, respectively), or (C) percentage body fat (>25% and >35% in men and women, respectively).

In all, 5,171 (97.6%) participants had available outcome data, 5,155 were censored at 1 year, and 129 (2.4%) participants were lost to follow-up. In the median 3.6 years (IQR 2.5 to 4.8 years) of follow-up, there were 145 events of the composite outcome of HF hospitalization or all-cause mortality. Higher SA quartiles were associated with a significantly lower risk for HF hospitalization or all-cause mortality (Table 3; Fig 2). The lean–malnourished (low BMI, low SA) and obese–malnourished (high BMI, low SA) groups had equally high crude event rates (30.1% and 29.5%; Table 3). Compared to the lean–well-nourished group (low BMI, high SA), the obese–malnourished group (high BMI, low SA) showed the highest multivariable-adjusted risk of the composite outcome (hazard ratio [HR] 2.49, 95% CI 1.43 to 4.34), followed by the lean–malnourished (HR 1.78, 95% CI 1.04 to 3.04, *p =* 0.034) and obese–well-nourished (HR 1.41, 95% CI 0.77 to 2.58; *p =* 0.27) groups (Table 3). Results were similar when analyzed by other anthropometric indices (waist circumference and body fat) (Tables 3 and 4). Formal analysis of the interaction between SA and anthropometric indices on the outcome did not reach significance (*p*_interaction_ > 0.05 for all; Table 3), possibly due to the low event rates.

**Table 4:** Summary of key findings on combined subgroups of obesity and nutrition.

**Lean–well-nourished**	**Lean–malnourished**
Youngest mean age (47.2 years)	Highest proportion of women (mean 57.9%)
Lowest percent body fat (mean 23.3%)	Normal average BMI
Lowest prevalence of comorbidities	Higher percent body fat (mean 24.5%)
**Lowest HF risk**	Higher prevalence of comorbidities (DM 7.5%)
	Higher NT-proBNP levels
	**Moderate HF risk**
**Obese–well-nourished**	**Obese–malnourished**
High waist circumference (mean 91.8 cm)	Oldest mean age (54.5 years)
Higher percent body fat (mean 29.2%)	High waist circumference (mean 91.7 cm)
Higher frequency of comorbidities (HTN 25.6%)	Highest percent body fat (mean 32%)
**Moderate HF risk**	Highest frequency of comorbidities (HTN 31%, DM 13%)
	Highest NT-proBNP levels
	**Highest HF risk**

DM, diabetes mellitus; HF, heart failure; HTN, hypertension; NT-proBNP, N-terminal pro B-type natriuretic peptide.

Similarly, results were consistent in the sensitivity analyses performed using other nutritional indices, PNI (S3 Table) and GLIM criteria (S5 and S6 Tables; S1 Fig). The obese–malnourished group (high BMI, GLIM-defined malnourishment) on average was older; had the largest waist circumference, the highest percentage body fat, and the highest burden of comorbidities (diabetes, 19.8%; hypertension, 36.6%; cardiovascular diseases, 15.3%); and had the least favorable diastolic indices and clinical outcomes, compared to the lean well-nourished group.

## Discussion

The present study aimed to elucidate the complex relationship between obesity, nutritional status, and cardiac outcomes in an Asian population. Among asymptomatic Asian individuals, we found that obese individuals (defined by BMI, waist circumference, or body fat) with poor nutritional status (defined by SA or PNI) had the highest burden of comorbidities, maladaptive cardiac remodeling, diastolic dysfunction, and the adverse composite outcome (HF hospitalization and mortality), much higher than in the lean–malnourished and obese–well-nourished groups (Table 4). The lean–well-nourished (low BMI, high SA) individuals had the most favorable metabolic health and outcomes.

Previous literature largely focused on examining the relationship between obesity or nutrition and cardiac outcomes in isolation [1,15,16,30], or used suboptimal measures of obesity and nutrition [14]. One study looking at the combined effect of obesity and nutrition showed that obese critically ill patients with malnutrition (versus well-nourished) had the poorest outcomes [14]. However, the study population consisted of critically ill patients (admitted in medical and surgical intensive care units) only, and heavily relied on BMI to define obesity [14], limiting the generalizability of the findings. Other studies on nutrition and cardiac outcomes have primarily focused on older or diseased populations (e.g., geriatric, with HF) [13,15,16] or utilized inconsistent measures of nutrition [15,16]. Of note, malnourished status (low SA) in our study was associated with increased HF events in both lean and obese individuals, suggesting a prognostic role of protein-energy nutrition, even in the general population [31]. Numerous studies have demonstrated the prognostic impact of nutrition in HF among elderly individuals using a range of scores, including SA [15,16], PNI [12,32], Controlling Nutritional Status (CONUT) [32], and Geriatric Nutritional Risk Index (GNRI) [13].

Two prior community cohorts have evaluated the relationship between nutritional status (SA) and HF outcomes in older adults [15,16]. The Health ABC Study (*n =* 2,907; mean age 73.6 years) and the Cardiovascular Health Study (CHS) (*n =* 5,450; mean age ≥ 65 years) showed that low SA concentration (<40 and <35 g/L, respectively) was associated with 13% and 34% higher risk of HF, respectively [15,16]. Notably, our study adds that nutritional status was an independent predictor of HF outcomes, even in younger individuals (mean age 49.6 years) and with a higher SA cutoff (45 g/L). Of note, sub-analysis of CHS revealed that the higher risk with low SA was only evident among those aged 73 years and below [15].

### Nutritionally unhealthy obesity—A distinct obesity phenotype

The most important finding in our present study centers around characterizing the new obese–malnourished phenotype. We showed that obese–malnourished individuals from Asia were mostly women, with visceral obesity and a high comorbidity burden. Indeed, Asian individuals are predisposed towards visceral obesity, compared to white individuals, even at the same BMI [30]. A recent report identified that stunting, wasting, and thinness in women have decreased in parallel with an increase in the prevalence of overweight and obesity in low- and middle-income countries across Asia and sub-Saharan Africa [33]. Urbanization and industrialization in Asia have made food of low nutritional value easily accessible and affordable, whilst promoting a sedentary lifestyle, all contributing to the double burden of obesity and malnutrition [6].

Despite being a convenient measure, BMI falls short in distinguishing between the various elements of body composition, body fat distribution, and fluid accumulation and absolute weight gain [34]. It is therefore noteworthy that our results using waist circumference showed an even stronger association with outcomes (larger HRs) than BMI, suggesting that visceral adiposity could potentially be an instrumental driver of risk. Results were further supported by our results based on percentage body fat. Visceral adiposity, including cardiac steatosis or increased pericardial fat deposition, as well as underlying comorbidities (such as hypertension, diabetes, and dyslipidemia), promote pro-inflammatory conditions, ultimately contributing to cardiovascular dysfunction [2,35].

Beyond visceral adiposity, we found that malnourished status (versus being well-nourished) was associated with less favorable cardiac structural and functional remodeling, to a greater extent in obese (overall and visceral) than lean asymptomatic individuals. The obese–malnourished individuals had the highest LVMi, greater RWT, and reduced diastolic function. Notably, these individuals also had the highest NT-proBNP levels at baseline, compared to other groups. An increased NT-proBNP level is an established marker for atrial wall stress and remodeling [36]. Yet, numerous studies have shown obesity (indexed by BMI) to be associated with lower NT-proBNP levels despite being an independent risk factor for HF [21–23]. This surfaces as a unique challenge in diagnosing chronic HF in obese patients, especially in an emergency department setting [23]. Interestingly, the obese–malnourished individuals in our study exhibited higher NT-proBNP levels in parallel with reduced diastolic function, compared to all other groups. The potential relationship between NT-proBNP, BMI, and malnutrition should be explored further for early diagnosis and management of HF in obese individuals.

### Less favorable cardiac outcomes in obese–malnourished individuals

Among asymptomatic individuals in our study, the obese–malnourished individuals were at markedly higher risk of the composite outcome, compared to all other subgroups. Our findings are consistent with those of Robinson and colleagues, who showed that among 6,518 critically ill patients, obese individuals (BMI > 30 kg/m^2^) with protein-energy malnutrition (assessed by dietitians) were at a 67% higher risk of 90-day mortality, compared to their well-nourished counterparts [14]. Physiologically, SA protects against oxidative stress and ischemic damage [8,9,11]. With the interplay between low SA levels and obesity pathology (pro-inflammatory signaling, excessive visceral adiposity, and obesity-related comorbidities), cardiac maladaptation ensues, potentially leading to HFpEF [37,38]. Indeed, in 1,677 HFpEF patients in the TOPCAT (Aldosterone Antagonist Therapy for Adults with Heart Failure and Preserved Systolic Function) trial, moderate to severe risk of malnutrition (GNRI < 92) was associated with a 34% higher adjusted risk of cardiovascular death, compared to those without the risk for malnutrition [13].

### Limitations and strengths

Anthropometric and echocardiographic data were not available beyond the baseline visit. We could not utilize the standardized set of diagnostic characteristics recommended by the Academy of Nutrition and Dietetics and the American Society for Parenteral and Enteral Nutrition (ASPEN) [39] to define adult malnutrition. Clinical data were not available during follow-up, restricting anthropometric and echocardiographic measurements as single-time-point measures at baseline. Findings reported are from a single-center study in Taiwan.

Nonetheless, our study is novel in comprehensively assessing the association of obesity (using various anthropometric measures) and nutrition (using objective biological markers, SA, PNI, and GLIM criteria) with cardiac characteristics and outcomes in a general population. SA concentration is mainly determined by rates of synthesis, protein degradation, and body losses and may serve as a measure of protein-calorie malnutrition [7–9]. PNI, an alternative nutritional marker that incorporates total lymphocyte count as part of its scoring together with SA, represents combined nutritional–inflammatory status and has also been shown to provide prognostic information [11,31,40,41], including in HF [31]. Other strengths of our current work include the detailed echocardiographic analyses, which demonstrated key cardiac morphological (left atrial and left ventricular) and subclinical functional changes in various combined subgroups of obesity and nutritional status, as well as the robust associations with the composite outcome.

### Conclusion

In this cohort study among asymptomatic Asian individuals, we observed that obese–malnourished individuals have the highest comorbidity burden, maladaptive cardiac remodeling, and the least favorable cardiac outcomes. Our findings suggest that the double burden of obesity and malnutrition in our Asian general community exists, and that healthy lifestyle programs and policies to promote nutritional health and reduce obesity are warranted in this region.

## Supporting information

S1 Checklist STROBE (DOCX)

S1 Fig Kaplan–Meier estimates of the composite outcome in subgroups of malnutrition defined by GLIM criteria. (DOCX)

S1 Table Baseline demographics and echocardiography information of study participants according to waist circumference and serum albumin categories. (DOCX)

S2 Table Baseline demographics and echocardiography information of study participants according to body fat composition and serum albumin categories. (DOCX)

S3 Table Association of subgroups of BMI and PNI with the composite outcome (heart failure hospitalization and all-cause mortality) (*n* = 156). (DOCX)

S4 Table Association of echocardiography indices with serum albumin concentrations and BMI, as individual variables. (DOCX)

S5 Table Baseline and echocardiographic characteristics of participants classified by GLIM-recommended malnutrition criteria. (DOCX)

S6 Table Association of subgroups of malnutrition (defined by GLIM criteria) with the composite outcome (heart failure hospitalization and all-cause mortality) (*n* = 145). (DOCX)

## Abbreviations

BSA: body surface area
eGFR: estimated glomerular filtration rate
GLIM: Global Leadership Initiative on Malnutrition
GNRI: Geriatric Nutritional Risk Index
HF: heart failure
HFpEF: heart failure with preserved ejection fraction
HDL-C: high-density lipoprotein cholesterol
HR: hazard ratio
hs-CRP: high-sensitivity C-reactive protein
LAVi: left atrial volume index
LV: left ventricular
LVEF: left ventricular ejection fraction
LVH: left ventricular hypertrophy
LVMi: left ventricular mass index
NT-proBNP: N-terminal pro B-type natriuretic peptide
PNI: prognostic nutritional index
RWT: relative wall thickness
SA: serum albumin
TDI: tissue Doppler imaging
