# Correction: Associations of obesity and malnutrition with cardiac remodeling and cardiovascular outcomes in Asian adults: A cohort study

**DOI:** 10.1371/journal.pmed.1003784

**Published:** 2021-09-13

**Authors:** Shih-Chieh Chien, Chanchal Chandramouli, Chi-In Lo, Chao-Feng Lin, Kuo-Tzu Sung, Wen-Hung Huang, Yau-Huei Lai, Chun-Ho Yun, Cheng Huang, Hung-I Yeh, Ta-Chuan Hung, Chung-Lieh Hung, Carolyn S. P. Lam

In the Methods and findings subsection of the Abstract, the first sentence is incorrect.

The correct sentence is: We examined 5,300 consecutive asymptomatic Asian participants who were recruited in a cardiovascular health screening program (mean age 49.6 ± 11.4 years, 64.8% male) between June 2009 to December 2012.

In the Study setting and population subsection of the Methods, the last sentence is incorrect.

The correct sentence is: Informed consent was waived for study participants. This study complied with the Declaration of Helsinki (S1 Checklist STROBE).
