# Supplementary material for: Associations of obesity and malnutrition with cardiac remodeling and cardiovascular outcomes in Asian adults: A cohort study
Source: PLoS Med. 2021 Jun 1;18(6):e1003661. doi: 10.1371/journal.pmed.1003661 (PMC8205172; doi:10.1371/journal.pmed.1003661)

S1 Fig: Kaplan-meier estimates of composite outcomes between subgroups of malnutrition defined by GLIM criteria.


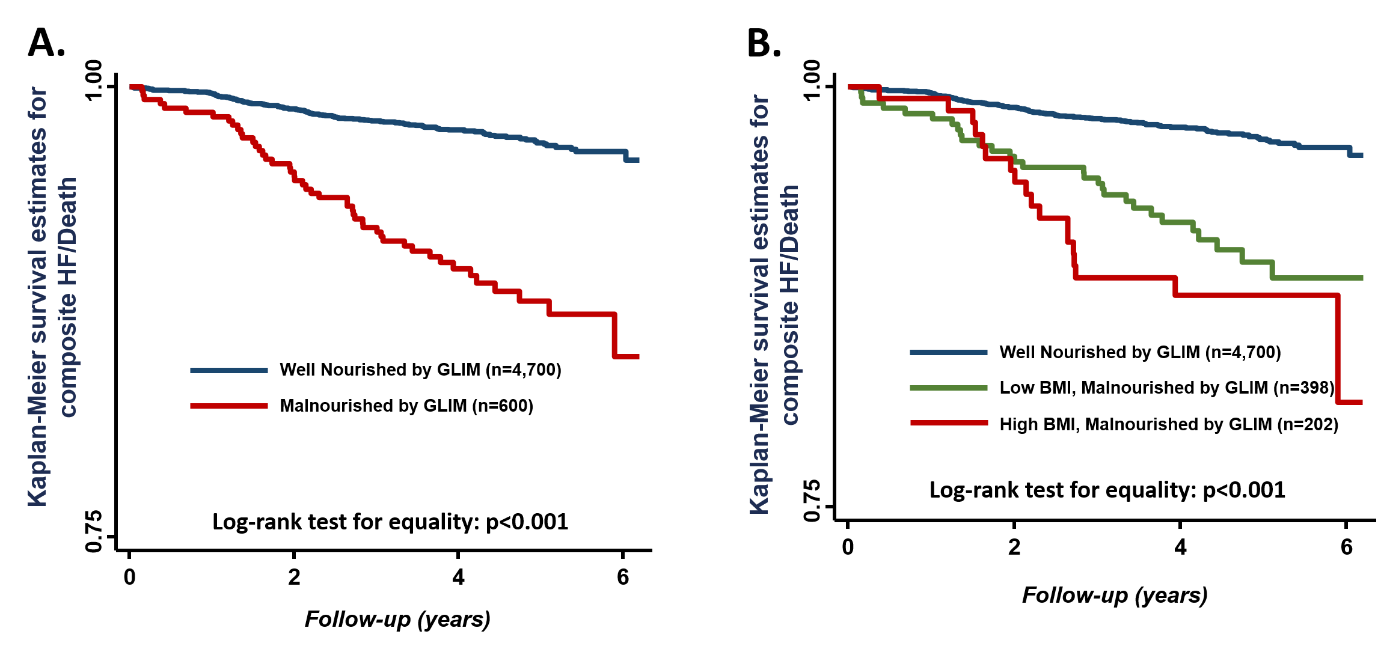

Supplement: S1 Fig — (DOCX) [file pmed.1003661.s002.docx]
