# Supplementary material for: Associations of obesity and malnutrition with cardiac remodeling and cardiovascular outcomes in Asian adults: A cohort study
Source: PLoS Med. 2021 Jun 1;18(6):e1003661. doi: 10.1371/journal.pmed.1003661 (PMC8205172; doi:10.1371/journal.pmed.1003661)
Supplement: S1 Table — (DOCX) [file pmed.1003661.s003.docx]

**SUPPLEMENTS**

**S1 Table: Baseline demographics and echocardiography Information of study participants according to waist and serum albumin categories**

| **Variables** | **Lean- well nourished** | **Obese- well nourished** | **Lean-malnourished** | **Obese-malnourished** | **p value** |
| --- | --- | --- | --- | --- | --- |
|  | **WC≤80,90, SA≥45** | **WC>80,90, SA≥45** | **WC≤80,90, SA<45** | **WC>80,90, SA<45** |  |
| ***Total N (n=5348)*** | *2503 (46.8%)* | *992 (18.5%)* | *1228 (23%)* | *625 (11.7%)* |  |
| ***Demographic Data*** |  |  |  |  |  |
| Age, years | 46.5±10.5 | 50.1±11.2***** | 52.0±11.1***^＃^** | 55.7±12.1***^＃†^** | <0.001 |
| Male gender, % | 1850 (73.9%) | 723 (72.9%) | 627 (51.1%) | 265 (42.4%) | <0.001 |
| Body mass index, kg/m^2^ | 23.1±2.53 | 27.8±3.30***** | 22.9±2.68**^＃^** | 27.5±3.89***^†^** | <0.001 |
| Systolic blood pressure, mmHg | 121.0±16.1 | 129.4±17.1***** | 119.9±17.5**^＃^** | 127.9±18.7***^†^** | <0.001 |
| Heart rate, beats/min | 67.7±11.1 | 68.7±11.4 | 66.2±11.4***^＃^** | 67.9±12.1**^†^** | <0.001 |
| Body fat | 23.6±4.91 | 30.8±6.02***** | 24.3±6.01***^＃^** | 32.8±7.83***^＃†^** | <0.001 |
| Waist circumference | 79.5±7.23 | 94.3±7.28***** | 77.7±7.59***^＃^** | 92.4±8.50***^＃†^** | <0.001 |
| Fat mass | 15.3±3.93 | 24.4±6.79***** | 14.8±4.43**^＃^** | 23.9±8.44***^†^** | <0.001 |
| Fat free mass | 49.2±7.84 | 54.5±10.0***** | 45.7±8.32***^＃^** | 48.7±10.2**^＃†^** | <0.001 |
| Hypertension, % | 318 (12.7%) | 298 (30%) | 179 (14.6%) | 202 (32.3%) | <0.001 |
| Diabetes, % | 106 (4.2%) | 92 (9.3%) | 92 (7.5%) | 83 (13.3%) | <0.001 |
| Cardiovascular disease, % | 120 (4.8%) | 90 (9.1%) | 100 (8.1%) | 73 (11.7%) | <0.001 |
| Smoking, % | 226 (9%) | 109 (11%) | 166 (13.5%) | 62 (9.9%) | <0.001 |
| Exercise, % | 313 (12.5%) | 137 (13.8%) | 189 (15.4%) | 91 (14.6%) | 0.09 |
| ***Laboratory Data and Biomarkers*** |  |  |  |  |  |
| White blood count | 6.02±1.53 | 6.60±1.70***** | 5.90±1.69**^＃^** | 6.40±1.65***^†^** | <0.001 |
| Fasting glucose, mg/dl | 99.2±15.9 | 108.1±27.8***** | 98.9±23.8**^＃^** | 101.1±21.9***^†^** | <0.001 |
| eGFR, mL/min/1.73m^2^ | 88.6±15.6 | 86.5±16.9***** | 90.4±19.6**^＃^** | 88.3±21.2 | <0.001 |
| Total cholesterol | 201.9±35.8 | 206.8±36.9***** | 197.2±39.2***^＃^** | 201.4±35.5**^＃^** | <0.001 |
| Triglyceride | 126.1±83.3 | 172.1±191.9***** | 119.1±131.7**^＃^** | 147.5±88.2***^＃†^** | <0.001 |
| LDL-c | 130.5±32.8 | 135.5±33.6***** | 124.0±33.0***^＃^** | 130.4±31.9**^＃†^** | <0.001 |
| HDL-c | 55.2±14.9 | 47.8±12.8***** | 57.1±16.1***^＃^** | 51.4±14.9***^＃†^** | <0.001 |
| Total protein | 7.55±0.37 | 7.58±0.37 | 7.20±0.41***^＃^** | 7.31±0.41***^＃†^** | <0.001 |
| Serum GPT | 27.7±20.1 | 40.1±31.1***** | 24.6±23.3***^＃^** | 31.5±30.2***^＃†^** | <0.001 |
| NT-proBNP | 33.6±34.7 | 39.9±52.0 | 72.6±222.8***^＃^** | 69.8±110.1***^＃^** | <0.001 |
| CRP | 0.15±0.33 | 0.26±0.35***** | 0.21±0.42***^＃^** | 0.30±0.37***^†^** | <0.001 |
| ***Echocardiography*** |  |  |  |  |  |
| IVS, mm | 8.85±1.08 | 9.50±1.21***** | 8.89±1.15**^＃^** | 9.41±1.17***^†^** | <0.001 |
| LVPW, mm | 8.85±0.98 | 9.47±0.97***** | 8.86±1.05**^＃^** | 9.42±1.09***^†^** | <0.001 |
| IVSi, mm/m^2^ | 4.81±0.62 | 4.67±0.67***** | 5.04±0.67***^＃^** | 4.90±0.71***^＃†^** | <0.001 |
| LVPWi, mm/m^2^ | 4.81±0.57 | 4.65±0.58***** | 5.04±0.65***^＃^** | 4.90±0.67***^＃†^** | <0.001 |
| LVIDD, mm | 46.2±3.57 | 48.0±3.50***** | 46.0±3.76**^＃^** | 47.6±3.29***^†^** | <0.001 |
| LVIDS, mm | 29.0±2.85 | 30.2±28.8***** | 28.8±3.00**^＃^** | 29.9±3.13***^†^** | <0.001 |
| LV EDV, ml | 74.6±13.8 | 81.9±14.2***** | 73.7±14.1**^＃^** | 79.6±13.7***^＃†^** | <0.001 |
| LE VSV, ml | 28.0±7.10 | 31.1±8.45***** | 27.4±7.30**^＃^** | 29.8±7.62***^＃†^** | <0.001 |
| LV EDVi, ml/m^2^ | 40.3±6.42 | 40.1±6.30 | 41.7±6.91***^＃^** | 41.3±6.62***^＃^** | <0.001 |
| LV ESVi, ml/m^2^ | 15.1±3.39 | 15.2±3.94 | 15.5±3.75***** | 15.4±3.66 | 0.01 |
| LVEF, % | 62.6±5.31 | 62.4±5.76 | 62.9±5.59 | 62.8±5.58 | 0.09 |
| LV mass m, gm/m^2^ | 137.0±30.2 | 159.9±33.5***** | 136.9±33.0**^＃^** | 155.8±33.7***^†^** | <0.001 |
| LV mass index, gm/m^2^ | 74.0±14.7 | 78.0±14.6***** | 77.1±16.2***** | 80.6±16.3***^＃†^** | <0.001 |
| LV mass index (Ht^2.7^), gm/m^2^ | 34.5±7.43 | 39.8±8.48***** | 36.9±8.51***^＃^** | 42.5±9.87***^＃†^** | <0.001 |
| Deceleration time, ms | 204.0±87.5 | 206.9±38.7 | 203.6±39.6 | 209.8±41.2 | 0.19 |
| IVRT, ms | 88.5±14.1 | 91.4±15.7***** | 89.7±15.6 | 92.1±17.4***^†^** | <0.001 |
| TDI-e’ (average), cm/sec | 9.83±2.38 | 8.26±2.13***** | 9.17±2.39***^＃^** | 7.94±2.22***^＃†^** | <0.001 |
| TDI-s’ (average), cm/sec | 8.57±1.57 | 8.10±1.54***** | 8.09±1.53***** | 7.75±1.43***^＃†^** | <0.001 |
| E/A ratio | 1.30±0.43 | 1.09±0.37***** | 1.26±0.45**^＃^** | 1.08±.044***^†^** | <0.001 |
| E/e’ (average) | 7.27±2.16 | 8.46±2.64***** | 8.24±2.76***** | 9.20±2.90***^＃†^** | <0.001 |
| Tau | 38.2±8.26 | 40.5±9.44***** | 41.1±10.1***** | 42.5±10.3***^＃†^** | <0.001 |
| TR velocity, m/sec | 2.06±0.29 | 2.09±0.33***** | 2.16±0.35***^＃^** | 2.23±0.34***^＃†^** | <0.001 |
| LAV (max), ml | 28.0±10.3 | 36.6±13.8***** | 30.3±12.4***^＃^** | 36.0±13.3***^†^** | <0.001 |
| LAVi, ml/m^2^ | 15.1±5.31 | 17.8±6.42***** | 17.1±6.81***^＃^** | 18.7±6.84***^†^** | <0.001 |

Abbreviations: GCS, global circumferential strain; GLS, global longitudinal strain; LDL, low-density lipoprotein; HDL, high-density lipoprotein; eGFR, Estimated Glomerular Filtration Rate, EDV, end-diastolic volume; EF, ejection fraction; TDI, Tissue Doppler imaging; TR: tricuspid regurgitation.

* p value<0.05 as compared with lean-well nourished^, ＃^ p value<0.05 as compared with obese-well nourished, † p value<0.05 as compared with lean-malnourished
