# Supplementary material for: Associations of obesity and malnutrition with cardiac remodeling and cardiovascular outcomes in Asian adults: A cohort study
Source: PLoS Med. 2021 Jun 1;18(6):e1003661. doi: 10.1371/journal.pmed.1003661 (PMC8205172; doi:10.1371/journal.pmed.1003661)
Supplement: S2 Table — (DOCX) [file pmed.1003661.s004.docx]

**S2 Table: Baseline demographics and echocardiography Information of study participants according to body fat composition and serum albumin categories**

| **Variables** | **Lean- well nourished** | **Obese- well nourished** | **Lean-malnourished** | **Obese-malnourished** | **p value** | **p (trend)** |
| --- | --- | --- | --- | --- | --- | --- |
|  | **BF≤25,35, SA≥45** | **BF>25,35, SA≥45** | **BF≤25,35, SA<45** | **BF>25,35, SA<45** |  |  |
| ***Total N (n=5136)*** | *2161 (42%)* | *1205 (23.5%)* | *1241 (24.2%)* | *529 (10.3%)* |  |  |
| ***Demographic Data*** |  |  |  |  |  |  |
| Age, years | 47.6±11.0 | 47.4±10.4 | 52.8±11.6**^*＃^** | 53.7±11.4**^*＃^** | <0.001 | <0.001 |
| Male gender, % | 1450 (67.1%) | 1042 (86.5%) | 563 (45.4%) | 301 (56.9%) | <0.001 | <0.001 |
| Body mass index, kg/m^2^ | 22.8±2.47 | 27.4±3.05**^*^** | 22.8±2.46**^＃^** | 28.1±3.62**^*＃†^** | <0.001 | <0.001 |
| Systolic blood pressure, mmHg | 120.6±16.2 | 128.0±16.6**^*^** | 119.7±17.8**^＃^** | 128±17.7**^*†^** | <0.001 | <0.001 |
| Heart rate, beats/min | 67.3±11.1 | 69.1±11.2**^*^** | 65.9±11.2**^*＃^** | 68.0±12.2**^†^** | <0.001 | 0.47 |
| Body fat | 22.8±4.68 | 30.6±5.34**^*^** | 24.1±5.46**^*＃^** | 34.4±7.83**^*＃†^** | <0.001 | <0.001 |
| Waist circumference | 79.5±7.91 | 91.5±8.02**^*^** | 78.9±8.38**^＃^** | 92.0±10.1**^*†^** | <0.001 | <0.001 |
| Fat mass | 14.5±3.33 | 23.9±6.06**^*^** | 14.6±3.64**^＃^** | 25.7±8.25**^*＃†^** | <0.001 | <0.001 |
| Fat free mass | 48.9±8.59 | 54.0±8.38**^*^** | 45.6±8.44**^*＃^** | 49.5±10.1**^＃†^** | <0.001 | <0.001 |
| Hypertension, % | 292 (13.5%) | 302 (25.1%) | 201 (16.2%) | 162 (30.6%) | <0.001 | <0.001 |
| Diabetes, % | 111 (5.1%) | 79 (6.6%) | 96 (7.7%) | 66 (12.5%) | <0.001 | <0.001 |
| Cardiovascular disease, % | 112 (5.2%) | 91 (7.6%) | 105 (8.5%) | 57 (10.8%) | <0.001 | <0.001 |
| Smoking, % | 167 (7.7%) | 167 (13.9%) | 148 (11.9%) | 77 (14.6%) | <0.001 | <0.001 |
| Exercise, % | 273 (12.6%) | 173 (14.4%) | 194 (15.6%) | 82 (15.5%) | 0.07 | 0.01 |
| ***Laboratory Data and Biomarkers*** |  |  |  |  |  |  |
| White blood count | 5.89±1.49 | 6.72±1.66**^*^** | 5.83±1.65**^＃^** | 6.57±1.67**^*†^** | <0.001 | <0.001 |
| Fasting glucose, mg/dl | 98.2±15.9 | 106.2±26.1**^*^** | 98.7±22.3**^＃^** | 106.3±26.1**^*†^** | <0.001 | <0.001 |
| eGFR, mL/min/1.73m^2^ | 88.6±16.1 | 86.9±15.2**^*^** | 89.7±19.2**^＃^** | 88.4±19.9 | 0.001 | 0.39 |
| Total cholesterol | 200.3±36.0 | 208.6±35.9**^*^** | 196.8±39.4**^＃^** | 203.0±34.1**^＃†^** | <0.001 | 0.52 |
| Triglyceride | 120.4±90.4 | 174.3±102.6**^*^** | 119.7±131.7**^＃^** | 150.5±84.6**^*＃†^** | <0.001 | 0.03 |
| LDL-c | 128.3±32.6 | 138.5±32.8**^*^** | 123.6±33.3**^*＃^** | 132.8±30.4**^*＃†^** | <0.001 | 0.81 |
| HDL-c | 56.2±15.2 | 46.8±11.3**^*^** | 57.5±16.3**^＃^** | 49.5±13.1**^*＃†^** | <0.001 | <0.001 |
| Total protein | 7.51±0.37 | 7.62±0.36**^*^** | 7.20±0.41**^*＃^** | 7.34±0.35**^*＃†^** | <0.001 | <0.001 |
| Serum GPT | 25.4±17.6 | 41.8±31.0**^*^** | 23.1±18.7**^*＃^** | 36.1±37.2**^*＃†^** | <0.001 | <0.001 |
| NT-proBNP | 38.2±42.1 | 30.0±37.6 | 73.0±178.0**^*＃^** | 69.3±227.1**^*＃^** | <0.001 | <0.001 |
| CRP | 0.15±0.34 | 0.24±0.32**^*^** | 0.20±0.41**^*^** | 0.32±0.38**^*＃†^** | <0.001 | <0.001 |
| ***Echocardiography*** |  |  |  |  |  |  |
| IVS, mm | 8.83±1.10 | 9.45±1.12**^*^** | 8.87±1.16**^＃^** | 9.50±1.16**^*†^** | <0.001 | <0.001 |
| LVPW, mm | 8.84±1.02 | 9.40±0.90**^*^** | 8.84±1.07**^＃^** | 9.50±0.99**^*†^** | <0.001 | <0.001 |
| IVSi, mm/m^2^ | 4.85±0.64 | 4.65±0.61**^*^** | 5.05±0.67**^*＃^** | 4.83±0.66**^＃†^** | <0.001 | <0.001 |
| LVPWi, mm/m^2^ | 4.85±0.59 | 4.62±0.52**^*^** | 5.03±0.64**^*＃^** | 4.84±0.61**^＃†^** | <0.001 | <0.001 |
| LVIDD, mm | 46.2±3.73 | 47.8±3.2**^*^** | 46.1±3.73**^＃^** | 47.8±3.15**^＃†^** | <0.001 | <0.001 |
| LVIDS, mm | 29.0±3.03 | 30.0±2.74**^*^** | 28.7±2.96**^＃^** | 30.1±3.11**^＃†^** | <0.001 | <0.001 |
| LV EDV, ml | 74.3±14.2 | 81.3±13.4**^*^** | 73.8±14.2**^＃^** | 80.6±12.9**^＃†^** | <0.001 | <0.001 |
| LE VSV, ml | 27.9±7.67 | 30.7±7.23**^*^** | 27.2±7.34**^＃^** | 30.4±2.27**^＃†^** | <0.001 | 0.001 |
| LV EDVi, ml/m^2^ | 40.6±6.58 | 39.8±5.93**^*^** | 41.8±6.88**^*＃^** | 41.0±6.26**^＃^** | <0.001 | 0.001 |
| LV ESVi, ml/m^2^ | 15.2±3.69 | 15.0±3.29 | 15.4±3.75**^＃^** | 15.4±3.47 | 0.03 | 0.04 |
| LVEF, % | 62.7±5.41 | 62.4±5.44 | 63.3±5.45**^＃^** | 62.5±5.58**^†^** | 0.001 | 0.8 |
| LV mass m, gm/m^2^ | 136.5±31.7 | 157.2±30.4**^*^** | 136.7±33.1**^＃^** | 158.7±3.15**^＃†^** | <0.001 | 0.001 |
| LV mass index, gm/m^2^ | 74.4±15.3 | 76.9±13.5**^*^** | 77.1±16.1**^*^** | 80.4±15.2**^*＃†^** | <0.001 | 0.001 |
| LV mass index (Ht^2.7^), gm/m^2^ | 34.6±7.81 | 38.7±7.88**^*^** | 36.9±8.48**^*＃^** | 42.7±9.42**^*＃†^** | <0.001 | 0.001 |
| Deceleration time, ms | 204.4±92.9 | 204.8±38.5**^*^** | 204.0±38.3 | 208.8±43.0 | 0.58 | 0.25 |
| IVRT, ms | 88.7±14.4 | 90.8±14.9 | 89.6±15.2 | 93.3±18.1**^*＃†^** | <0.001 | 0.001 |
| TDI-e’ (average), cm/sec | 9.79±2.45 | 8.63±2.10**^*^** | 9.13±2.39**^*＃^** | 7.98±2.26**^*＃†^** | <0.001 | <0.001 |
| TDI-s’ (average), cm/sec | 8.52±1.59 | 8.28±1.50**^*^** | 8.09±1.51**^*＃^** | 7.77±1.42**^*＃†^** | <0.001 | <0.001 |
| E/A ratio | 1.29±0.44 | 1.13±0.36**^*^** | 1.25±0.45**^*＃^** | 1.08±0.41**^*†^** | <0.001 | <0.001 |
| E/e’ (average) | 7.41±2.31 | 7.94±2.39**^*^** | 8.29±2.71**^*＃^** | 9.06±2.84**^*＃†^** | <0.001 | <0.001 |
| Tau | 38.7±8.63 | 39.4±8.75 | 41.2±10.0**^*＃^** | 42.7±10.4**^*＃†^** | <0.001 | <0.001 |
| TR velocity, m/sec | 2.08±0.30 | 2.06±0.32 | 2.17±0.34**^*＃^** | 2.22±0.33**^*＃†^** | <0.001 | <0.001 |
| LAV (max), ml | 27.8±10.5 | 35.2±13.0**^*^** | 29.9±11.3**^*＃^** | 37.1±13.6**^*＃†^** | <0.001 | <0.001 |
| LAVi, ml/m^2^ | 15.2±5.44 | 17.2±6.12**^*^** | 16.9±6.23**^*^** | 18.8±6.80**^*＃†^** | <0.001 | <0.001 |

Abbreviations: BF, body fat (%); GCS, global circumferential strain; GLS, global longitudinal strain; LDL, low-density lipoprotein; HDL, high-density lipoprotein; eGFR, Estimated Glomerular Filtration Rate, EDV, end-diastolic volume; EF, ejection fraction; TDI, Tissue Doppler imaging; TR: tricuspid regurgitation.

* p value<0.05 as compared with lean-well nourished^, ＃^ p value<0.05 as compared with obese-well nourished, † p value<0.05 as compared with lean-malnourished

Sex-specific cut-offs for body fat were used (25 and 35% for men and women respectively)
