# Supplementary material for: Associations of obesity and malnutrition with cardiac remodeling and cardiovascular outcomes in Asian adults: A cohort study
Source: PLoS Med. 2021 Jun 1;18(6):e1003661. doi: 10.1371/journal.pmed.1003661 (PMC8205172; doi:10.1371/journal.pmed.1003661)
Supplement: S3 Table — (DOCX) [file pmed.1003661.s005.docx]

**S3: Association of subgroups of BMI and PNI on composite outcomes (heart failure hospitalization and all cause mortality) (n=156)**

|  | **Lean-well nourished** | **Obese-well nourished** | **Lean-malnourished** | **Obese-malnourished** |
| --- | --- | --- | --- | --- |
| **Subgroups of PNI and BMI Indices** | **BMI≤25, PNI≥55** | **BMI>25, PNI≥55** | **BMI≤25, PNI<55** | **BMI>25, PNI<55** |
| *Univariate,* HR [95% CI] | (Reference) | 1.15 [0.63, 2.12], p=0.64 | 1.81 [1.09, 3.00], p=0.021 | 3.50 [2.10, 5.82], p<0.001 |
| *Multi-variate,* HR [95% CI] | (Reference) | 1.14 [0.56, 2.32], p=0.034 | 1.45 [0.81, 2.58], p=0.21 | 2.21 [1.22, 4.01], p=0.009 |
|  |  |  |  |  |
| **Subgroups of PNI and waist circumferences** | **WC≤80,90, PNI≥55** | **WC>80,90, PNI≥55** | **WC≤80,90, PNI<55** | **WC>80,90, PNI<55** |
| *Univariate,* HR [95% CI] | (Reference) | 1.41 [0.77, 2.58], p=0.27 | 1.72 [1.08, 2.76], p=0.024 | 4.34 [2.72, 6.93], p<0.001 |
| *Multi-variate,* HR [95% CI] | (Reference) | 1.31 [0.66, 2.61], p=0.44 | 1.42 [0.81, 2.49], p=0.22 | 2.47 [1.37, 4.43], p=0.002 |
|  |  |  |  |  |
| **Subgroups of PNI and body fat percentage** | **BF≤25,35, PNI≥55** | **BF>25,35, PNI≥55** | **BF≤25,35, PNI<55** | **BF>25,35, PNI<55** |
| *Univariate,* HR [95% CI] | (Reference) | 1.37 [0.73, 2.59], p=0.33 | 2.53 [1.51, 4.23], p<0.001 | 3.11 [1.73, 5.59], p<0.001 |
| *Multi-variate,* HR [95% CI] | (Reference) | 1.43 [0.71, 2.87], p=0.32 | 1.75 [1.00, 3.05[, p=0.048 | 2.12 [1.12, 3.99], p=0.02 |

*Multi-variate* - adjusted for age, sex, systolic blood pressure, heart rate, fasting glucose, high-density lipoprotein cholesterol, total cholesterol, hypertension, diabetes, cardiovascular disease, and estimated glomerular filtration rate (eGFR).
