# Supplementary material for: Associations of obesity and malnutrition with cardiac remodeling and cardiovascular outcomes in Asian adults: A cohort study
Source: PLoS Med. 2021 Jun 1;18(6):e1003661. doi: 10.1371/journal.pmed.1003661 (PMC8205172; doi:10.1371/journal.pmed.1003661)
Supplement: S4 Table — (DOCX) [file pmed.1003661.s006.docx]

**S4 : Association of echocardiography indices with serum albumin concentrations and BMI, as individual variables.**

|  | **Albumin Tertile Groups** | | |  | **Multi-variate model** | | |
| --- | --- | --- | --- | --- | --- | --- | --- |
|  | **Tertile 1**  **(≤ 4.4 mg/dL)** | **Tertile 2**  **(4.5-4.6 mg/dL)** | **Tertile 3**  **(≧ 4.7 mg/dL)** | ***P (trend)*** | **SA (per 1 mg/dL increase)** | **BMI (per 1 kg/m^2^ increase)** | **P_interaction_**  **BMI*SA** |
| ***Echocardiography*** |  |  |  |  |  |  |  |
| IVS, mm | 9.1 (1.3) | 9.1 (1.3) | 9.0 (1.2) | <0.001 | -0.19 (-0.32, -0.05) | 0.08 (0.07, 0.09) | **0.03** |
| Lean (BMI≤25 kg/m^2^) |  |  |  |  | *-0.11 (-0.27, 0.04)* |  |  |
| Obese (BMI>25 kg/m^2^) |  |  |  |  | *-0.32 (-0.54, -0.09)* |  |  |
| LVPW, mm | 9.1 (1.2) | 9.0 (1.2) | 9.0 (1.1) | 0.01 | -0.18 (-0.30, -0.06) | 0.08 (0.07, 0.09) | **0.007** |
| Lean (BMI≤25 kg/m^2^) |  |  |  |  | *-0.11 (-0.25, 0.04)* |  |  |
| Obese (BMI>25 kg/m^2^) |  |  |  |  | *-0.36 (-0.56, -0.15)* |  |  |
| IVSi, mm/m^2^ | 5.0 (0.8) | 4.9 (0.7) | 4.7 (0.7) | <0.001 | -0.08 (-0.16, -0.01) | — | — |
| LVPWi, mm/m^2^ | 5.0 (0.7) | 4.8 (0.7) | 4.7 (0.6) | <0.001 | -0.08 (-0.15, -0.01) | — | — |
| LVIDD, mm | 46.8 (3.8) | 46.8 (3.8) | 47.0 (3.5) | 0.13 | -0.90 (-1.27, -0.53) | 0.31 (0.28, 0.34) | NS |
| LVIDS, mm | 29.3 (3.2) | 29.4 (3.0) | 29.6 (2.9) | 0.001 | -0.53 (-0.85, -0.21) | 0.23 (0.20, 0.25) | NS |
| LV EDV, ml | 75.8 (14.2) | 76.2 (14.6) | 77.6 (13.9) | <0.001 | -3.92 (-5.26, -2.48) | 1.17 (1.06, 1.28) | NS |
| LE VSV, ml | 28.3 (7.5) | 28.6 (7.6) | 29.3 (7.6) | <0.001 | -1.57 (-2.39, -0.76) | 0.50 (0.44, 0.56) | NS |
| LV EDVi, ml/m^2^ | 41.6 (6.8) | 40.5 (6.8) | 40.0 (6.3) | <0.001 | -2.36 (-3.11, -1.60) | — | — |
| LV ESVi, ml/m^2^ | 15.5 (3.7) | 15.2 (3.4) | 15.1 (3.7) | 0.002 | -0.98 (-1.40, -0.55) | — | — |
| LVEF, % | 62.9 (8.6) | 62.6 (5.5) | 62.4 (5.7) | 0.002 | 0.12 (-0.54, 0.78) | -0.07 (-0.12, -0.02) | NS |
| LV mass, gm/m^2^ | 145.3(38.0) | 144.6 (37.9) | 143.8 (34.2) | 0.41 | -8.89 (-12.4, -5.42) | 3.31 (3.05, 3.58) | 0.002 |
| Lean (BMI≤25 kg/m^2^) |  |  |  |  | *-9.34 (-13.3, -5.36)* |  |  |
| Obese (BMI>25 kg/m^2^) |  |  |  |  | *-14.0 (-20.2, -7.76)* |  |  |
| LV mass index, gm/m^2^ | 79.3 (18.8) | 76.5 (17.1) | 74.0 (15.8) | <0.001 | -5.49 (-7.32, -3.66) | — | — |
| LV mass index (Ht^2.7^), gm/m^2^ | 39.4 (10.8) | 37.1 (9.6) | 35.3 (8.8) | <0.001 | -3.23 (-4.23, -2.22) | — | — |
| LVH, % | 201 (11.0%) | 110 (6.0%) | 55 (3.4%) | <0.001 | — | — | — |
| RWT | 0.39 (0.05) | 0.39 (0.06) | 0.38 (0.05) | <0.001 | -0.01 (-0.01, 0.01) | 0.001 (0.0004, 0.001) | NS |
| Geometry, % |  |  |  | <0.001 | — |  | — |
| Normal | 1352 (73.7%) | 1422 (77.8%) | 1324 (81.0%) |  |  |  |  |
| Concentric Remodelling | 282 (15.4%) | 296 (16.2%) | 256 (15.7%) |  |  |  |  |
| Eccentric Hypertrophy | 100 (5.5%) | 29 (1.6%) | 23 (1.4%) |  |  |  |  |
| Concentric Hypertrophy | 101 (5.5%) | 81 (4.4%) | 32 (2.0%) |  |  |  |  |
| Deceleration time, ms | 206.0 (40.7) | 205.2 (39.5) | 203.3 (38.1) | 0.13 | -5.77 (-10.4, -1.19) | 0.10 (-0.24, 0.45) | NS |
| IVRT, ms | 91.0 (16.3) | 89.9 (15.2) | 89.3 (14.3) | 0.002 | 0.44 (-1.29, 2.16) | 0.08 (-0.05, 0.21) | NS |
| TDI-e’ (average), cm/sec | 8.75 (2.40) | 9.14 (2.34) | 9.63 (2.45) | <0.001 | 1.38 (1.16, 1.62) | -0.09 (-0.10, -0.07) | **0.017** |
| Lean (BMI≤25 kg/m^2^) |  |  |  |  | 0.84 (0.49, 1.19) |  |  |
| Obese (BMI>25 kg/m^2^) |  |  |  |  | 1.43 (1.13, 1.73) |  |  |
| TDI-s’ (average), cm/sec | 7.98 (1.51) | 8.23 (1.51) | 8.65 (1.60) | <0.001 | 0.25 (0.08, 0.42) | -0.03 (-0.05, -0.02) | NS |
| E/A ratio | 1.19 (0.45) | 1.22 (0.42) | 1.23 (0.42) | <0.001 | 0.09 (0.05, 0.13) | -0.01 (-0.02, -0.01) | NS |
| E/e’ (average) | 8.61 (2.89) | 7.92 (2.47) | 7.31 (2.26) | <0.001 | -0.51 (-0.77, -0,25) | 0.08 (0.06, 0.10) | **0.034** |
| Lean (BMI≤25 kg/m^2^) |  |  |  |  | -1.24 (-1.54, -0.93) |  |  |
| Obese (BMI>25 kg/m^2^) |  |  |  |  | -1.67 (-2.12, -1.22) |  |  |
| Tau | 41.6 (10.2) | 39.8 (9.0) | 37.9 (8.2) | <0.001 | -0.47 (-1.52, 0.59) | 0.21 (0.13, 0.29) | NS |
| TR velocity, m/sec | 2.20 (0.37) | 2.10 (0.34) | 2.07 (0.33) | <0.001 | -0.09 (-0.13, -0.05) | 0.02 (-0.01, 0.05) | NS |
| LAV (max), ml | 32.4 (12.7) | 31.1 (11.9) | 30.8 (13.2) | <0.001 | -3.27 (-4.50, -2.03) | 1.29 (1.20, 1.39) | NS |
| LAVi, ml/m^2^ | 17.7 (6.5) | 16.2 (5.7) | 15.5 (5.5) | <0.001 | -2.16 (-2.83, -1.49) | — | — |

Abbreviations: IVS, interventricular septal wall thickness, LV, left ventricular, LVPW, left-ventricular posterior wall thickness, Ht^2.7^, indexed to height, LVIDD, left ventricular internal diameter in diastole, LVIDS, left ventricular internal diameter in systole, EDV, end diastolic volume, ESV, end systolic volume, EF, ejection fraction, BSA, body surface area, LVH, left ventricular hypertrophy, RWT, relative wall thickness, IVRT, interventricular relaxation time, TDI, tissue doppler imaging, e’, myocardial relaxation velocity, s’, -based peak systolic annular velocity, E/A, ratio of early (E) to late (A) ventricular filling velocities, E/e’, ratio of early mitral inflow velocity and mitral annular early diastolic velocity, tau, left ventricular diastolic time constant TR, tricuspid regurgitation velocity, LAV, left atrial volume, LAVi, left atrial volume index.

Multivariable analysis adjusted for age, sex, systolic blood pressure, heart rate, fasting glucose, high-density lipoprotein cholesterol, total cholesterol, hypertension, diabetes, cardiovascular disease, and estimated glomerular filtration rate (eGFR).
