# Supplementary material for: Associations of obesity and malnutrition with cardiac remodeling and cardiovascular outcomes in Asian adults: A cohort study
Source: PLoS Med. 2021 Jun 1;18(6):e1003661. doi: 10.1371/journal.pmed.1003661 (PMC8205172; doi:10.1371/journal.pmed.1003661)
Supplement: S5 Table — (DOCX) [file pmed.1003661.s007.docx]

**S5: Baseline and echocardiographic characteristics of participants classified by GLIM-recommended malnutrition criteria.**

|  | **Well-nourished**  n=4,700 | **Lean-malnourished**  n=398 | **Obese-malnourished**  n=202 | **p-value (ANOVA)** | **P _trend_** |
| --- | --- | --- | --- | --- | --- |
| ***Total N (n=5300)*** | *4700 (88.7%)* | *398 (7.5%)* | *202 (3.8%)* |  |  |
| ***Demographic Data*** |  |  |  |  |  |
| **Age, years** | **48.9±11.0** | **54.8±12.7^*^** | **54.9±12.3^*^** | <0.001 | <0.001 |
| Male gender, % | 3157 (67.2%) | 186 (46.7%) | 113 (55.9%) | <0.001 | <0.001 |
| Body mass index, kg/m^2^ | 24.5±3.47 | 21.3±2.05**^*^** | 29.0±3.83**^*＃^** | <0.001 | <0.001 |
| Systolic blood pressure, mmHg | 123.1±17.1 | 120.4±18.6**^*^** | 130.0±18.4**^*＃^** | <0.001 | <0.001 |
| Heart rate, beats/min | 67.4±11.3 | 68.0±11.8**^*^** | 70.9±13.2**^*＃^** | <0.001 | <0.001 |
| **Body fat** | **26.0±6.37** | **23.8±6.62^*＃^** | **34.8±10.40^*^** | <0.001 | <0.001 |
| Waist circumference | 83.7±9.73 | 76.6±7.88**^*^** | 93.7±10.22**^*＃^** | <0.001 | <0.001 |
| **Fat mass** | **18.0±6.48** | **13.5±4.25^*^** | **26.7±9.47^*＃^** | <0.001 | <0.001 |
| Fat free mass | 49.9±8.98 | 42.6±7.26* | 50.3±11.10**^＃^** | <0.001 | 0.571 |
| **Hypertension, %** | **843 (17.9%)** | **74 (18.6%)** | **74 (36.6%)** | <0.001 | <0.001 |
| **Diabetes, %** | **292 (6.2%)** | **37 (9.3%)** | **40 (19.8%)** | <0.001 | <0.001 |
| **Cardiovascular disease, %** | **297 (6.3%)** | **53 (13.3%)** | **31 (15.3%)** | <0.001 | <0.001 |
| Smoking, % | 461 (9.8%) | 72 (18.1%) | 28 (13.9%) | <0.001 | <0.001 |
| Exercise, % | 644 (13.7%) | 57 (14.3%) | 27 (13.4%) | 0.931 | 0.941 |
| ***Laboratory Data and Biomarkers*** |  |  |  |  |  |
| White blood count | 6.12±1.61 | 6.17±1.76 | 6.84±1.91**^*＃^** | <0.001 | <0.001 |
| Fasting glucose, mg/dl | 100.9±21.0 | 99.7±26.0 | 111.0±28.7**^*＃^** | <0.001 | <0.001 |
| eGFR, mL/min/1.73m^2^ | 88.4±16.9 | 89.5±20.4 | 90.1±23.0 | 0.224 | 0.188 |
| Total cholesterol | 201.9±36.1 | 202.8±44.9 | 197.6±38.4 | 0.253 | 0.117 |
| Triglyceride | 135.6±93.2 | 131.8±212.2 | 156.9±86.4**^*＃^** | 0.019 | 0.007 |
| LDL-c | 130.5±32.9 | 126.9±34.2 | 127.9±34.1**^*^** | 0.085 | 0.288 |
| HDL-c | 53.5±14.9 | 57.9±16.3**^*^** | 48.2±13.3**^*＃^** | <0.001 | <0.001 |
| Total protein | 7.45±0.40 | 7.34±0.44**^*^** | 7.36±0.41**^*^** | <0.001 | 0.003 |
| Serum GPT | 30.2±25.6 | 24.0±18.8**^*^** | 33.8±23.0**^＃^** | <0.001 | 0.051 |
| **Nt-ProBNP** | **41.6±59.0** | **90.5±269.2^*^** | **112.1±378.1^*^** | <0.001 | <0.001 |
| CRP | 0.16±0.28 | 0.51±1.07**^*^** | 0.53±0.50**^*^** | <0.001 | <0.001 |
| ***Echocardiography*** |  |  |  |  |  |
| IVS, mm | 9.03±1.30 | 8.88±1.21 | 9.81±1.44**^*＃^** | <0.001 | <0.001 |
| LVPW, mm | 9.00±1.19 | 8.82±1.16**^*^** | 9.82±1.16**^*＃^** | <0.001 | <0.001 |
| IVSi, mm/m^2^ | 4.81±0.73 | 5.25±0.82**^*^** | 4.93±0.78**^*^** |  |  |
| LVPWi, mm/m^2^ | 4.80±0.68 | 5.21±0.79**^*＃^** | 4.93±0.73**^*^** |  |  |
| LVIDD, mm | 46.9±3.70 | 45.5±3.86**^*^** | 48.7±3.17**^*＃^** | <0.001 | <0.001 |
| LVIDS, mm | 29.4±2.98 | 28.4±3.05**^*^** | 31.0±3.79**^*＃^** | <0.001 | <0.001 |
| LV EDV, ml | 76.8±14.2 | 70.4±13.7**^*^** | 82.0±13.4**^*＃^** | <0.001 | <0.001 |
| LE VSV, ml | 28.8±7.47 | 26.2±7.55**^*^** | 31.5±8.96**^*＃^** | <0.001 | <0.001 |
| LV EDVi, ml/m^2^ | 40.6±6.40 | 41.5±7.49**^*^** | 41.3±6.87 | 0.022 | 0.189 |
| LV ESVi, ml/m^2^ | 15.2±3.51 | 15.4±4.17 | 15.9±4.71 | 0.036 | 0.015 |
| LVEF, % | 62.6±5.46 | 63.0±5.99 | 61.9±6.95 | 0.096 | 0.087 |
| LV mass, gm/m^2^ | 144.4±36.5 | 133.5±32.9**^*^** | 171.5±40.4**^*＃^** | <0.001 | <0.001 |
| LV mass index, gm/m^2^ | 76.1±17.1 | 78.6±18.9**^*^** | 86.0±19.9**^*＃^** | <0.001 | <0.001 |
| LV mass index (Ht^2.7^), gm/m^2^ | 37.0±9.63 | 36.7±10.02 | 46.5±12.07**^*＃^** | <0.001 | <0.001 |
| LVH, % | 277 (5.9%) | 46 (11.6%) | 43 (21.3%) | <0.001 | <0.001 |
| RWT | 0.39±0.05 | 0.39±0.06 | 0.40±0.06**^*＃^** | <0.001 | <0.001 |
| Geometry, % |  |  |  |  |  |
| Normal | 3694 (78.6%) | 274 (68.8%) | 130 (64.4%) | <0.001 | <0.001 |
| Concentric Remodeling | 727 (15.5%) | 78 (19.6%) | 29 (14.4%) |  |  |
| Eccentric Hypertrophy | 112 (2.4%) | 24 (6.0%) | 16 (7.9%) |  |  |
| Concentric Hypertrophy | 165 (3.5%) | 22 (5.5%) | 27 (13.4%) |  |  |
| Deceleration time, ms | 204.6±39.2 | 208.4±41.2 | 206.0±42.7 | 0.175 | 0.614 |
| IVRT, ms | 89.9±15.1 | 91.9±16.0**^*^** | 91.0±17.9 | 0.029 | 0.331 |
| TDI-e’ (average), cm/sec | 9.28±2.43 | 8.38±2.23**^*^** | 7.69±1.81**^*＃^** | <0.001 | <0.001 |
| TDI-s’ (average), cm/sec | 8.33±1.55 | 7.92±1.63**^*^** | 7.77±1.51**^*^** | <0.001 | <0.001 |
| E/A ratio | 1.22±0.42 | 1.18±0.47 | 1.05±0.43**^*＃^** | <0.001 | <0.001 |
| E/e’ (average) | 7.83±2.48 | 8.88±3.14**^*^** | 9.51±3.64**^*＃^** | <0.001 | <0.001 |
| Tau | 39.4±8.91 | 43.4±12.19**^*^** | 41.9±10.68**^*^** | <0.001 | <0.001 |
| TR velocity, m/sec | 2.11±0.34 | 2.24±0.41**^*^** | 2.23±0.40**^*^** | <0.001 | <0.001 |
| LAV (max), ml | 30.9±11.7 | 28.5±11.2**^*^** | 38.0±13.9**^*＃^** | <0.001 | <0.001 |
| LAVi, ml/m^2^ | 16.3±5.85 | 16.9±6.84 | 19.1±7.27**^*＃^** | <0.001 | <0.001 |

Abbreviations: IVS, interventricular septal wall thickness, LV, left ventricular, LVPW, left-ventricular posterior wall thickness, Ht^2.7^, indexed to height, LVIDD, left ventricular internal diameter in diastole, LVIDS, left ventricular internal diameter in systole, EDV, end diastolic volume, ESV, end systolic volume, EF, ejection fraction, BSA, body surface area, LVH, left ventricular hypertrophy, RWT, relative wall thickness, IVRT, interventricular relaxation time, TDI, tissue doppler imaging, e’, myocardial relaxation velocity, s’, -based peak systolic annular velocity, E/A, ratio of early (E) to late (A) ventricular filling velocities, E/e’, ratio of early mitral inflow velocity and mitral annular early diastolic velocity, tau, left ventricular diastolic time constant TR, tricuspid regurgitation velocity, LAV, left atrial volume, LAVi, left atrial volume index.
