# Supplementary material for: Associations of obesity and malnutrition with cardiac remodeling and cardiovascular outcomes in Asian adults: A cohort study
Source: PLoS Med. 2021 Jun 1;18(6):e1003661. doi: 10.1371/journal.pmed.1003661 (PMC8205172; doi:10.1371/journal.pmed.1003661)
Supplement: S6 Table — (DOCX) [file pmed.1003661.s008.docx]

**S6: Association of subgroups of malnutrition (defined by GLIM criteria) on composite outcomes (heart failure hospitalization and all-cause mortality) (n=145)**

|  | **Well-nourished**  n=4,700 | **Lean-malnourished**  n=398 | **Obese-malnourished**  n=202 |
| --- | --- | --- | --- |
| *Number of crude events (n)* | 96 (2.0%) | 29 (7.3%) | 20 (9.9%) |
| *Univariate,* HR [95% CI] | (Reference) | 3.38 [2.26, 5.08], p<0.001 | 4.64 [2.88, 7.49], p<0.001 |
| *Multi-variate,* HR [95% CI] | (Reference) | 1.78 [1.09, 2.92], p=0.021 | 2.75 [1.56, 4.82], p<0.001 |

Abbreviations: HR, hazards ratio, CI, confidence interval.

*Multi-variate* - adjusted for age, sex, systolic blood pressure, heart rate, fasting glucose, high-density lipoprotein cholesterol, total cholesterol, hypertension, diabetes, cardiovascular disease, and estimated glomerular filtration rate (eGFR).
